# Random and non-random variation in flower colour along an urban–rural gradient in the introduced mustard *Hesperis matronalis*

**DOI:** 10.1093/aob/mcag035

**Published:** 2026-02-17

**Authors:** Katherine G Maunder, Kaitlyn Dawson, Lauryn Joslin, Lucas Eckert, Marlene M Kraml, Chloë Dean-Moore, Christopher G Eckert

**Affiliations:** Department of Biology, Queen’s University, Kingston, Ontario K7L 3N6 Canada; Department of Ecology and Evolutionary Biology, University of Toronto, Toronto, Ontario M5S 3B2 Canada; Department of Biology, Queen’s University, Kingston, Ontario K7L 3N6 Canada; Department of Biology, Queen’s University, Kingston, Ontario K7L 3N6 Canada; Department of Biology, McGill University, Montréal, Quebec H3A 1B1 Canada; Department of Biology, Queen’s University, Kingston, Ontario K7L 3N6 Canada; Department of Biology, Queen’s University, Kingston, Ontario K7L 3N6 Canada; Department of Biology, Queen’s University, Kingston, Ontario K7L 3N6 Canada

**Keywords:** Brassicaceae, flower colour, genetic drift, *Hesperis matronalis*, natural selection, non-indigenous plants, polymorphism, seed predation

## Abstract

**Background and Aims:**

Urbanization can alter the interplay of evolutionary processes such as natural selection and genetic drift, but these effects will depend on the biology and history of a species. To explore the influences of drift and selection in an urban context, we investigated patterns of flower colour variation among stands of the introduced ornamental mustard *Hesperis matronalis* along an urban–rural gradient in eastern Ontario, Canada.

**Methods:**

We surveyed 136 naturalized stands of *H. matronalis* over three generations, and for each stand estimated the diversity of the three colour morphs (white, pink, purple), the number of reproductive plants and the degree of urbanization based on night sky brightness.

**Key Results:**

Flower colour morph diversity increased with both stand size and urbanization, which is consistent with effects of genetic drift during colonization combined with multiple introductions of this horticultural plant in urban areas. However, the frequency and fixation of the purple morph systematically increased towards the rural end of the gradient. Although lifetime seed production did not vary among morphs, pre-dispersal seed predation by a recent adventive weevil was higher in the purple morph, particularly in rural areas. Estimated seed production in the absence of predation possibly suggests a previous fitness advantage for the purple and pink morphs in rural areas and for the white morph in urban areas.

**Conclusions:**

Random variation in flower colour diversity may be influenced by stochastic processes and colonization history, while systematic variation in colour morph frequencies may reflect past fitness differences among morphs that have been recently erased by seed predation.

## INTRODUCTION

Human activity is changing the evolutionary dynamics of many species, especially in urban habitats (Johnson and Munshi-South, 2017; Alberti *et al*., 2020). Urbanization can expose species to novel environments and thus alter the form and strength of natural selection. This may create clines in ecologically important traits along urban–rural gradients (e.g. Thompson *et al*., 2016; Santangelo *et al*., 2022). However, urban areas can also differ from surrounding rural areas in the quantity, structure and isolation of appropriate habitats (Miles *et al*., 2019), which may alter neutral processes such as genetic drift and gene flow. While it can be challenging to identify the relative effects of selective and neutral processes (Santangelo *et al*., 2018), cities provide an opportunity to study the contemporary interplay of these evolutionary forces in widely available ecosystems. Beyond adding to our fundamental understanding of evolutionary processes, teasing apart the eco-evolutionary consequences of urbanization is also critical for effective urban planning and conservation (Alberti *et al*., 2020).

Current experimental evidence suggests that while all major evolutionary processes can be influenced by urbanization, the direction and magnitude of these effects will vary between species depending on their ecology and thus how individual species respond to urbanization (Miles *et al*., 2019). For many native species, urbanization fragments available habitat which can lead to (1) decreases in population size, which can also increase the loss of variation due to genetic drift, and (2) increases in the spatial isolation of populations thereby restricting the replenishment of variation via gene flow (Noël and Lapointe, 2010; Bartlewicz *et al*., 2015; Munshi-South *et al*., 2016; Rivkin and Johnson, 2022). However, the opposite pattern may be observed in species that are well suited to urban environments and in species that are dispersed by human activity (Cadotte *et al*., 2017; Miles *et al*., 2019). For example, non-native plants have a long history of being introduced to urban areas through horticulture (Reichard and White, 2001; Mack and Erneberg, 2002; Harris *et al*., 2009; van Kleunen *et al*., 2018) and their subsequent range expansion after introduction is often assisted by human activity (Kowarik, 2003; Dehnen-Schmutz *et al*., 2007; McLean *et al*., 2017). In such cases, populations may be larger and less spatially isolated in urban areas compared to rural areas and thus better able to maintain genetic variation. In addition, any stochastic reduction in genetic variation associated with founder effects during individual introductions can be offset by multiple introductions of a species (Barney, 2006; Roman and Darling, 2007; Dlugosch and Parker, 2008; Pairon *et al*., 2010; Vicente *et al*., 2021), which may be particularly common in urban areas (Lu *et al*., 2022; Mairal *et al*., 2022). Thus, urban populations of introduced horticultural plants can, in some cases, serve as genetically diverse launching sites for the invasion of surrounding rural areas. During subsequent range expansion, sequential founder effects will probably decrease genetic diversity in populations further from the introduction sites (Austerlitz *et al*., 1997; Slatkin and Excoffier, 2012), thereby creating a pattern of declining population genetic diversity from urban to rural areas. In this way, clines in phenotypic and genetic diversity along urban–rural gradients may be the result of neutral, non-selective forces (Barney, 2006; Pairon *et al*., 2010; Miles *et al*., 2019).

Urbanization can also change the abiotic and biotic environment that a species experiences, which may lead to novel selective pressures and hence phenotypic differentiation between urban and rural populations (Johnson and Munshi-South, 2017). Altered selection may be inferred if the spatial patterns of phenotypic variation along an urban–rural gradient deviate from those expected under the sole influence of neutral processes. For instance, in an introduced species whose range is expanding and whose dispersal is assisted by human activity (e.g. by the air and soil displacement of moving cars), neutral processes can decrease genetic and phenotypic variation in rural populations compared to urban populations (as outlined above), but this reduction in variation should be random with no bias towards any particular allele or phenotype (Hedrick, 2000; but see Santangelo *et al*., 2018). In contrast, a consistent change in the frequency of an allele or phenotype (e.g. a decrease in variation is always associated with an increase in the frequency of a particular allele or phenotype) may suggest that selection varies along the urban–rural gradient. For example, consistently high frequencies of wing-less queen morphs in the ant species *Myrmecina graminicola* in areas of high urbanization and habitat fragmentation suggest selection for lower dispersal in more urban areas (Finand *et al*., 2024).

In this study, we investigated the role of stochastic and selective processes affecting the flower colour polymorphism of introduced *Hesperis matronalis* L. (Brassicaceae, dame’s rocket). Discrete phenotypic polymorphisms have provided excellent opportunities to study the interplay of neutral and selective forces in natural populations (Schemske and Bierzychudek, 2007; Barrett, 2019). If a polymorphism has a relatively simple genetic basis, then population variation in discrete phenotypic ‘morphs’ may reflect genetic variation at the underlying trait loci (Joron *et al*., 2006; Kingsley *et al*., 2009). Studies of flower colour polymorphisms (both those within and between populations) were central to the debate over the prevalence of neutral versus selective processes influencing phenotypic variation in natural populations (Epling and Dobzhansky, 1942; Schemske and Bierzychudek, 2001). Yet since then, few studies have investigated the processes influencing patterns of colour variation within populations across space or time (Sapir *et al*., 2021). Flower colour is often thought to be under selection (Faegri and van der Pijl, 1979), although definitive evidence of this is usually lacking for most species studied (Rausher, 2008). Nevertheless, selection is often suspected because flower colour can influence the attraction and foraging behaviours of pollinators, which can lead to changes in gene transmission and ultimately individual fitness (Waser and Price, 1981; Meléndez-Ackerman and Campbell, 1998). In *H. matronalis*, flowers are pollinated during the day and night (Francis *et al*., 2009) by a variety of insects (bees, butterflies, moths and syrphid flies) that reportedly do not exhibit a flower colour preference (Majetic et al., 2007, 2008). Flower colour can also influence how plants respond to a wide variety of biotic and abiotic factors (Strauss and Whittall, 2007; Caruso *et al*., 2019; Koski and Galloway, 2020) because many of the pigments responsible for petal colour, such as anthocyanins, also play a role in stress tolerance and herbivore resistance (reviewed in Lev-Yadun and Gould, 2008; Naing and Kim, 2021). For instance, reduced herbivory on morphs with higher anthocyanin pigmentation has been documented in *Raphanus sativus* (petal colour variation, Irwin *et al*., 2003) and *Quercus coccifera* (leaf colour variation, Karageorgou and Manetas, 2006). In *H. matronalis*, the composition of herbivore fauna and whether any herbivores respond to flower colour is unknown. Recently, an *H. matronalis* specialist weevil (*Ceutorhynchus inaffectatus* Gyllenhal, 1837, Curculionidae) from the native European range (Larsen *et al*., 1992) has been reported in southern Ontario (Pentinsaari *et al*., 2019) but it is not known whether it has any impact on introduced populations of *H. matronalis* or if it attacks plants non-randomly with respect to flower colour.

We quantified the pattern of flower colour variation in *H. matronalis* and surveyed the frequency of three distinct flower colour morphs in 136 stands (clusters of plants) across an urban to rural gradient in southeastern Ontario, Canada. To explore the relative influence of neutral and selective processes on flower colour variation in introduced stands of *H. matronalis*, we tested whether flower colour variation deviated from established theoretical expectations of a neutral cline. We tested five predictions consistent with a selectively neutral cline in flower colour variation. (1) Flower colour morph diversity (a measure of the richness and evenness of colour morph occurrence) should be higher in larger stands because larger stands are less vulnerable to losses of genetic diversity from drift. (2) Flower colour morph diversity should be greater in more urban stands (independent of the effect of stand size predicted above) due to assumed higher levels of independent horticultural introduction in areas with more human activity. (3) Variation in morph diversity across generations should be lower in stands that maintain a larger average stand size and a more consistent stand size across generations (see Hedrick, 2000). (4) The random chance of a morph being fixed (or lost) within stands should be proportional (or inversely proportional) to the global frequency of that morph (Hedrick, 2000). Variation in colour morph diversity between stands across the urban–rural gradient should not coincide with a consistent increase or decrease in the frequency of any one morph. (5) Colour morphs should not differ in components or correlates of fitness.

Deviations from these five neutral predictions could indicate a possible role for selection. For example, if flower colour morph diversity decreases with increasing stand size (a departure from neutral prediction 1) this would be consistent with a potential decrease in morph diversity due to more efficient selection in larger stands. Additionally, any consistent and repeated change in morph frequencies (a departure from neutral prediction 4) may indicate fitness differences between morphs (a departure from neutral prediction 5). If non-neutral patterns are observed, we predict that there may be a consistent increase in the frequency of the purple morph in more urban areas and that purple morphs may have higher fitness. This prediction is based on the possibility that higher anthocyanin levels may deter herbivory and that there could be a higher abundance of the recently introduced specialist weevil in more urban areas.

## METHODS

### Study species

Native to Eurasia, *H. matronalis* is an herbaceous biennial (vegetative in its first year, reproductive in the second) or short-lived perennial that was introduced to urban North America as an ornamental garden plant beginning in the 1800s and has since become naturalized across southern Canada and the northern United States (Rousseau, 1968; Francis *et al*., 2009). The species is still widely sold across North America and naturalized populations are now particularly common in disturbed habitat along roads and railways, especially in urban areas (Francis *et al*., 2009). *Hesperis matronalis* seeds are generally assumed to not travel far from the parent plant since seed dispersal by wind and water is not very effective, but there have been some reports of seed dispersal by birds (Francis *et al*., 2009). Little is known about the seed bank of *H. matronalis* besides an unpublished report that it is probably weakly persistent (Francis *et al*., 2009). Populations of *H. matronalis* are often polymorphic for flower colour, although the pattern of phenotypic variation in flower colour has not been formally quantified. Currently the only available information on the possible genetic basis of flower colour in *H. matronalis* is from Majetic *et al.* (2007) who recognized two colour morphs, white and purple, and after a very limited number of hand crosses reported that the pattern of colour segregation was consistent with colour being determined by a single Mendelian locus with white dominant to purple. However, others have noted that intermediate pink phenotypes are common within most populations (Mitchell and Ankeny, 2001; Rothfels *et al*., 2002; Weeks and Frey, 2007; Francis *et al*., 2009), strongly suggesting that the inheritance of petal colour in this species is probably more complex.

Little is known about the selective agents that may potentially act on flower colour in *H. matronalis*. Majetic *et al.* (2007, 2008) reported that insects pollinating *H. matronalis* do not exhibit flower colour preferences and the floral scent composition, though variable and probably relevant to pollinators, is independent of colour. While *H. matronalis* does produce nectar as a pollen reward (Davis *et al*., 1998), the extent to which pollination is required for seed set is uncertain due to conflicting evidence that *H. matronalis* is self-compatible (Majetic *et al*., 2008) and possibly predominantly self-fertilizing (Susko and Clubb, 2008) or self-incompatible and obligately outcrossing (Mitchell and Ankeny, 2001; Weeks and Frey, 2007).

### Study sites

We located 136 naturalized stands of *H. matronalis* over a 1300-km^2^ area within a 40-km radius of Kingston, Ontario, Canada. We first surveyed these stands in June 2021 and returned the next two consecutive years, which amounts to three separate generations of flowering for this biennial species (Supplementary Data Appendix S1). The study area covered a gradient of human activity and contained stands that varied widely in census size. A stand was defined as a discrete group of plants separated by at least 100 m from other such groups (median distance was 500 m). We are using the term ‘stand’ instead of ‘population’ to acknowledge that while there is physical distance between stands, we cannot speak on whether they are genetically isolated. We accessed and surveyed 132 of these stands in June 2021, 112 stands in June 2022 and 116 stands in June 2023. Of these, 105 stands were sampled in all three years. Five sites were devoid of plants after 2021; seven sites had plants in 2021 and 2023 but not in 2022. For 19 stands, we could only gain access for one (12) or two (seven) of the sampling years.

### Geographical survey of flower colour variation

**Fig. 1.**
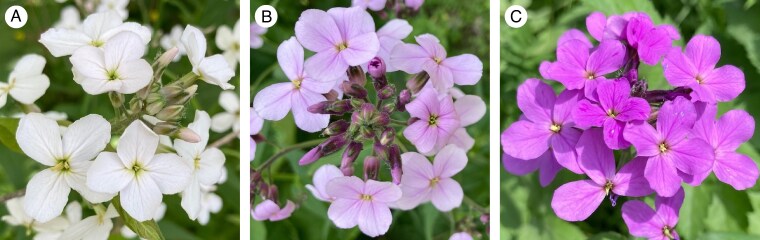
Flower colour variation in introduced stands of *Hesperis matronalis* from eastern Ontario, Canada. White morph flowers (A) were pure white with no hints of pink. Pink flowers (B) ranged from having obvious hints of pink, often in the centre of the flower, to petals that were predominantly pink with regions of darker pink. Purple flowers (C) were solid purple.

Previous work on flower colour variation in *H. matronalis* has classified flowers as either light or dark (Majetic *et al.*, 2007, 2008), though other researchers have noted considerable intermediate variation in flower colour (Mitchell and Ankeny, 2001; Rothfels *et al*., 2002; Weeks and Frey, 2007). To better understand the pattern of phenotypic variation, we performed spectral analysis on more than 1000 flowers from three large stands of *H. matronalis* in our study area. Although the distribution of flower colour was largely bimodal, there was enough intermediate flower colour variation to justify a three-morph classification to better capture phenotypic variation and consequently capture a better estimate of flower colour diversity (Supplementary Data Appendix S2). The results of this three-morph analysis are reported below, but we also ran a simplified two-morph analysis where flowers were classified as dark or light and the results were quite similar (Appendix S3). For each stand, we estimated flower colour morph frequencies by randomly sampling an average of 96 plants (range = 1–423) and scoring flowers as white (W), pink (P) or purple (V = violet) using a set of standard photographs to ensure consistency (Fig. 1). We sampled all plants in stands consisting of fewer than 100 plants and a random sample of 100 for larger stands (in a few populations more than 100 plants were sampled). For each stand, we directly counted the number of reproductive individuals (stand size, *N*) or for larger stands, we tallied plants in groups of 10 with a clicker counter while we surveyed the stand for flower colour variation.

### Quantifying human activity levels

We used night sky brightness as a proxy for the level of human activity around each sampled stand of *H. matronalis* (Sutton *et al*., 2001; Pun and So, 2012). While urbanization is associated with changes in many variables such as land use, pollution and the quantity of impermeable surfaces, we are specifically interested in how human activity (the presence and movement of humans) may be related to the population biology of *H. matronalis*. In our study region, there is a higher density of ornamental flower gardens in highly populated urban areas, and thus more possible sites for *H. matronalis* to be introduced. Night sky brightness can successfully estimate human population density, making it well-suited for our study (Sutton *et al*., 2001; Pun and So, 2012). Other aspects of human activity, such as agricultural land use, are not captured by night sky brightness but given the invasion history of *H. matronalis* as a garden escape (Francis *et al*., 2009), agriculture is probably not a primary influence on the species. We obtained zenith sky brightness data from Falchi *et al*.’s (2016) world atlas of artificial sky luminance and accessed these data through lightpollutionmap.info. The zenith sky brightness for each stand was reported in units of magnitude per square arc second (mag arcsec^−2^) at a spatial resolution of 1 km. Magnitudes are a measure of brightness and a smaller value indicates a brighter object. Zenith sky brightness ranges from 16 mag arcsec^−2^ for the brightest urban skies (highest human population density) to 22 mag arcsec^−2^ for the very darkest skies (very low human density). Among the *H. matronalis* stands we sampled, zenith sky brightness ranged across much of this scale (19.15–21.89) and correlated strongly and negatively with distance from Kingston city centre (Pearson *r* = −0.82, *n* = 136, *P* < 0.0001). The spatial resolution of the zenith sky brightness data was also appropriate for our study because 99 % of all the pairwise distances between the stands that we sampled were >1 km. To make zenith sky brightness easier to interpret in terms of human activity, we multiplied these values by −1 and refer to this as ‘night sky brightness’ (NSB). As a result, there is a positive correlation between night sky brightness and human activity. For example, a rural stand could have an NSB value of −21, while an urban stand, where light pollution is greater, could have an NSB value of −19.

### Comparing fitness correlates and seed predation among colour morphs

For a subsample of 22 stands out of the 132 surveyed in 2021 (see Supplementary Data Appendix S1 for a map), we tagged 10–20 randomly chosen plants per colour morph. Then in mid-August, the above-ground portion of the 215 plants that could be relocated with intact tags were harvested when fruits were mature but seeds not yet released. After drying plants to constant mass at 70 °C, we counted the number of mature fruits (each containing ≥1 filled seed) as well as the number of flowers that failed to develop fruits (indicated by persistent petioles). Above-ground vegetative plant size was estimated by total dry stem mass, because only about 30 % of plants still had some of their leaves at this stage. We estimated flower number as the sum of fruit number and the number of fruits that did not develop. From each plant, we randomly selected five fruits and counted the number of filled seeds in each. Total seed production was estimated for each plant as the product of fruit number and the average number of filled, undamaged seeds per fruit. Many seeds were destroyed by the pre-dispersal seed predator *Ceutorhynchus inaffectatus* (D.J. Ensing, T. Nelson & C.G. Eckert, unpublished), so to estimate the number of seeds that could have potentially matured in the absence of seed predation, we counted the indentations left by developing seeds in the silique septum. From these data, we estimated the proportion of seeds destroyed by *C. inaffectatus*. We also estimated potential seed production in the absence of seed predation as the product of fruit number and mean potential seeds per fruit.

### Statistical analyses

#### Neutral prediction 1 & 2: morph diversity should increase with stand size and NSB

We measured flower colour morph diversity of each stand using Shannon’s diversity index (*H*′) calculated using the *diversity* function in the vegan package (v.2.6-2; https://CRAN.R-project.org/packag=vegan) for the R statistical environment (v.4.2.1, R Core Team, 2022), which was used for all analyses. *H*′ is particularly appropriate when sample sizes are variable and sometimes small (Morris *et al*., 2014) and is correlated strongly with other measures of morph diversity such as morph evenness (e.g. *r* ≥ +0.99, all *P* < 0.0001; Barrett *et al*., 1989). The distribution of stand size (*N*) was strongly right-skewed, so we log_10_-transformed this variable for analysis (log_10_*N*). To test the predictions that flower colour morph diversity would be higher in larger stands (higher log_10_*N*, prediction 1) and stands in areas with higher human activity (higher NSB, prediction 2), we fit variation in *H*′ to a linear model including log_10_*N*, NSB and their interaction as predictors using the *lm* function in base R. The response and predictor variables were scaled by their means and standard deviations to generate standardized partial regression coefficients that could be compared between predictors and years. We tested the significance of each predictor using backward stepwise elimination with likelihood ratio tests. Because a different number of sites were sampled in each year, we performed this analysis for each year separately. For all analyses, we ensured that residuals were normally distributed and independent from predicted values. We calculated the variance inflation factor (VIF) for both predictors using the *vif* function in the car package (v.3.1-1, https://CRAN.R-project.org/package=car) to confirm that any collinearity between predictors did not complicate the interpretation of partial regression coefficients. We used added-variable plots to graphically display the direct effect of NSB and log_10_*N* on *H*′ while controlling the effect of the other predictor and accounting for the weak collinearity between predictors.

#### Neutral prediction 3: morph diversity should vary less when stand size is high and generationally consistent

Variation in colour morph diversity across generations was evaluated using the 105 stands surveyed in all three years. To measure variation, we used the standard deviation of *H*′ across years instead of the coefficient of variation in *H*′ because the standard deviation was less correlated with mean *H*′ across years (s.d. *r* = −0.31, CV *r* = −0.85). We fit variation in the standard deviation of *H*′ to a linear model with NSB, mean log_10_*N* and the standard deviation of log_10_*N* as predictors, and evaluated the significance of predictors and displayed the data graphically as above.

#### Neutral prediction 4a: the probability of fixation/loss should be proportional to the global frequency of a morph

Population genetic theory predicts that small populations will tend to fix (or lose) alleles more frequently than larger populations (Hedrick, 2000), so we compared log_10_*N* between fixed and polymorphic stands using *t*-tests (*t.test* function in base R). A fixed/monomorphic stand is defined here as a population where all sampled plants are of the same colour morph. In the specific case of *H. matronalis*, we also might expect urban stands to maintain colour morph diversity more frequently than rural stands due to a possible higher chance of multiple introductions in urban areas. Thus, we also compared NSB between fixed and polymorphic stands using *t*-tests (*t.test* function in base R). Theory also predicts that the probability of an allele being randomly fixed in a population is proportional to its global frequency (Hedrick, 2000). This theoretical prediction assumes that polymorphic populations lack population structure and that fixed populations are independently derived from this source. We tested the neutral prediction that the frequency of *H. matronalis* stands stochastically fixed for a colour morph should be proportional to the mean frequency of that morph in polymorphic stands using a chi-squared test with a *P* value derived from Monte Carlo simulation (*chisq.test* function in base R). Conversely, we tested whether the frequency of stands that had lost a particular morph were inversely proportional to the frequency of that morph in polymorphic stands.

#### Neutral prediction 4b: morph diversity should not be consistently shaped by the frequency of a particular morph

We also investigated if, contrary to neutral predictions, the frequency of any one flower colour morph consistently increased or decreased with human activity and stand size. To do this we fit morph frequencies as a multinomial response variable to a generalized linear model using the *multinom* function in the nnet R package (v.7.3-17, https://CRAN.R-project.org/package=nnet). We included NSB, log_10_*N* and their interaction as fixed predictors and evaluated significance with stepwise likelihood ratio tests as above. Because the interaction was significant for all years, we illustrated trends graphically by plotting model predictions made using the *predict* function in base R over the full range of NSB and at the 25-percentile, median and 75-percentile of log_10_*N*. We determined how well the model predicted morph frequencies for each morph separately by regressing observed frequencies over predicted frequencies and calculating the *r*^2^ value and *P* value from an *F*-test.

We found a consistent change in colour morph frequencies with both NSB and log_10_*N* involving an increase in the frequency of the purple morph in stands that were smaller and in areas of lower human activity (see Results). This result led us to test whether human activity and stand size influenced morph diversity beyond just their influence on the frequency of the purple morph. To do this, we used the morph frequency values predicted by our multinomial regression to calculate ‘expected morph diversity’ (*H*′_pred_) and included this as a covariate in the previously described linear models. All together this allowed us to examine the effect of human activity (NSB) and stand size (log_10_*N*) on observed morph diversity (*H*′) while accounting for the impact of systematic variation in individual morph frequencies on diversity. We performed significance tests, checked assumptions and made graphical displays of the results as described above for the original regression analysis.

#### Neutral prediction 5: morphs should not differ in fitness

We used linear models to compare fitness components and seed predator damage among colour morphs in a subsample of 22 stands. Each model contained variation in a fitness component as the response variable; colour morph, NSB and their interaction as fixed effects; and stand as a random effect. The significance of the models was evaluated with stepwise likelihood ratio tests as above. We fit log_10_-transformed above-ground stem dry mass to a linear mixed-effects model using the *lmer* function in the lme4 R package (v.1.1-30, https://CRAN.R-project.org/package=lme4). Because flower number, fruit number, seed number and potential seed number per plant are over-dispersed count data, we used generalized linear mixed-effects models (GLMMs) with negative binomial errors fit using the *glmmTMB* function in the glmmTMB package (v.1.1.4, https://cran.r-project.org/package=glmmTMB). The negative binomial distribution is appropriate for count data and, unlike the Poisson distribution, includes a dispersion parameter. For the proportion of flowers setting fruit and the proportion of seeds destroyed by *C. inaffectatus* we used GLMMs with binomial errors with *glmmTMB*. We evaluated whether the residuals of each model were normally distributed using the *simulateResiduals* function and tested for over/underdispersion of residuals using the *testDispersion* function, both in the DHARMa package (v.0.4.6, https://cran.r-project.org/package=DHARMa). Residuals for fruit set were over dispersed, so we modelled a dispersion parameter using the *dispformula* argument in *glmmTMB*. Seeds per plant was modelled with a zero-inflation parameter applied to all observations using the *ziformula* argument.

## RESULTS

### Neutral predictions 1 & 2: morph diversity should increase with stand size and NSB [✓]

**Fig. 2.**
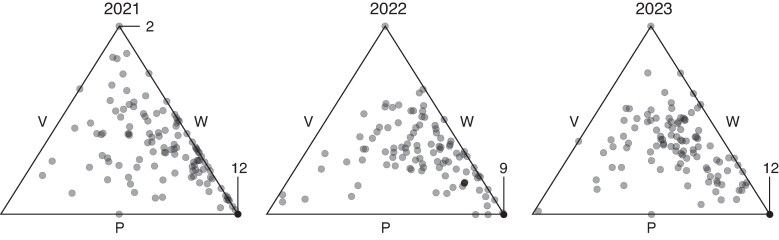
Variation in colour morph frequencies among stands of *Hesperis matronalis* in eastern Ontario, Canada. Each point represents the morph frequencies in a stand (132 stands in 2021, 112 in 2022, 116 in 2023). Colour morphs are white (W), pink (P) and purple (V) and the distance of a point from a morph’s side is proportional to the frequency of that morph in the stand. Points in the centre of the triangle have equal frequencies of all three morphs, those lying on a side lack that morph, and those lying on a vertex are fixed for the morph indicated on the opposite side. Lines and numbers indicate the number of points overlapping on a vertex.

**Fig. 3.**
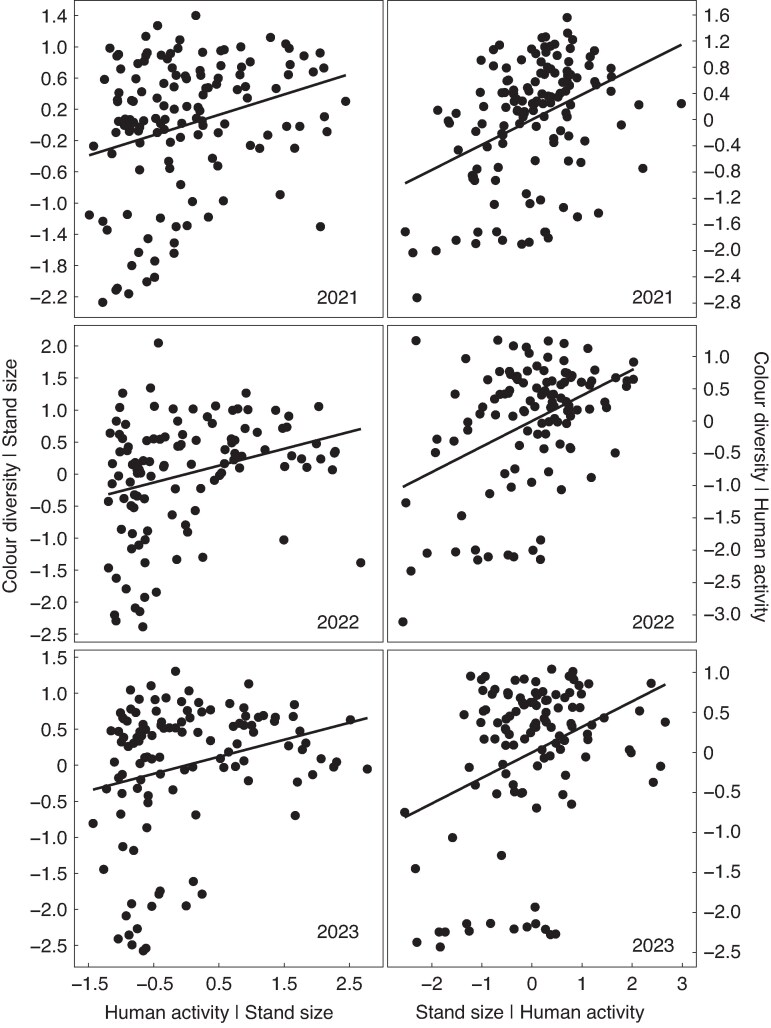
Added-variable plots showing the relations between flower colour morph diversity (*H′*) and both human activity (NSB) and stand size (log_10_*N*) among stands of *Hesperis matronalis* from eastern Ontario, Canada, sampled over three years (132 stands in 2021, 112 in 2022, 116 in 2023). Each point is a stand. The *y*-axis is residual *H*′ when the other predictor is held constant. The *x*-axis is the residual of the focal predictor when the other predictor is held constant. For example, plots on the left show the relationship between residual *H′* and residual human activity when stand size is held constant.

There was wide variation in the frequencies of the three flower colour morphs among stands in all years (Fig. 2). Flower colour morph diversity (*H*′) can vary from 0 to 1.10, where 0 indicates a monomorphic stand and 1.10 indicates a stand where all three morphs are equally frequent. In all three years *H*′ varied across almost the full range of possible values (2021: range = 0–1.09, mean = 0.71; 2022: range = 0–1.10, mean = 0.77; and 2023: range = 0–1.10, mean 0.81). As predicted, flower colour morph diversity increased significantly and independently with both stand size (log_10_*N*) and human activity (NSB) in all years (Table 1; Fig. 3) and the effects of these two predictors did not interact (all *P* > 0.21). While log_10_*N* and NSB correlated positively but weakly in all three years, the correlation was only significant for 2021 (2021: Pearson’s *r* = +0.35, *P* < 0.0001; 2022: *r* = +0.14, *P* = 0.13; 2023: *r* = +0.15, *P* = 0.11). Collinearity between predictors does not complicate the interpretation of the partial regression coefficients as the VIFs for all predictors were <1.1.

**Table 1.** Analyses of colour morph diversity (*H*′) among stands of *Hesperis matronalis*. In analysis A, linear models with human activity (NSB), stand size (log_10_*N*) and their interaction as predictors were used to explain variation in *H*′ (132 stands for 2021, 112 for 2022, 116 for 2023). Cells include *F*-tests of significance and standardized partial regression coefficients (*b*) when significant, or NA when that predictor was not included in that analysis. The effect of both human activity and stand size on *H*′ (Fig. 2) could result from a systematic increase in the frequency of the purple morph in smaller stands and in areas with low human activity. In analysis B we included ‘predicted morph diversity’ (*H*′_pred_) calculated from predicted morph frequencies (Fig. 3) as a covariate in the linear models to control for this effect. *H*′_pred_ was only a significant predictor of *H*′ in 2023 and this reduced the effect of both NSB and log_10_*N*. However, predicted morph diversity correlated with both of these other predictors, resulting in relatively high variance inflation factors (see Results).

	A			B		
Year	2021	2022	2023	2021	2022	2023
*r* ^2^	0.29	0.26	0.18	0.29	0.26	0.21
**Predictor**						
Human activity (NSB)	*b* = +0.261	*b* = +0.264	*b* = +0.236	*b* = +0.261	*b* = +0.264	*b* = +0.170
	*F*_1,129_ = 10.7	*F*_1,109_ = 10.0	*F*_1,113_ = 7.5	*F*_1,129_ = 10.7	*F*_1,109_ = 10.0	*F*_1,112_ = 3.5
	*P* = 0.0013	*P* = 0.0020	*P* = 0.0071	*P* = 0.0013	*P* = 0.0020	*P* = 0.064
Stand size (log_10_*N*)	*b* = +0.384	*b* = +0.396	*b* = +0.319	*b* = +0.384	*b* = +0.396	*b* = +0.251
	*F*_1,129_ = 23.3	*F*_1,109_ = 22.5	*F*_1,113_ = 13.7	*F*_1,129_ = 23.3	*F*_1,109_ = 22.5	*F*_1,112_ = 7.5
	*P* < 0.0001	*P* < 0.0001	*P* = 0.00033	*P* < 0.0001	*P* < 0.0001	*P* = 0.0070
NSB × log_10_*N*	–	–	–	NA	NA	NA
	*F*_1,128_ = 0.30	*F*_1,108_ = 1.56	*F*_1,112_ = 0.20			
	*P* = 0.58	*P* = 0.21	*P* = 0.66			
Predicted morph diversity (*H*′_pred_)	NA	NA	NA	–	–	*b* = +0.199
				*F*_1,128_ = 1.9	*F*_1,108_ = 0.42	*F*_1,112_ = 4.15
				*P* = 0.17	*P* = 0.52	*P* = 0.044

Among the 105 stands sampled in all three years, there was year to year consistency in both stand size and colour morph diversity, as *H*′ correlated strongly between years (Kendall’s coefficient of concordance *W* = +0.75, *P* < 0.0001) as did log_10_*N* (*W* = +0.80, *P* < 0.0001). The correlation between *H*′ and both NSB and log_10_*N* varied slightly between years but was always positive (Supplementary Data Appendix S4). On average, *H*′ varied among years and was significantly higher in 2023 than in the two previous years (mean ± 1s.e.: 2021 = 0.734 ± 0.031, 2022 = 0.768 ± 0.032, 2023 = 0.847 ± 0.029). This was associated with significantly higher stand size in 2023. For individual stands, the increase in flower colour morph diversity between 2023 and the two previous years was higher for stands in areas with lower human activity (lower NSB). This increase correlated positively but not significantly with the between-year increase in stand size and was not related to the year-to-year change in sample size (Appendix S5).

### Neutral prediction 3: morph diversity should vary less when stand size is high and is generationally consistent [✓]

Consistent with prediction 3, the among-generation variation in *H*′ (Supplementary Data Appendix S6) was lower in stands that were: (1) larger (mean of log_10_*N* across generations: standardized partial regression *b* = −0.31, *F*_1,101_ = 11.3, *P* = 0.0011), (2) less variable in size (s.d. of log_10_*N* across generations: *b* = +0.29, *F*_1,101_ = 10.1, *P* = 0.0019) and (3) in areas with higher human activity, though this effect was not significant (NSB: *b* = −0.13, *F*_1,101_ = 2.0, *P* = 0.16).

### Neutral prediction 4a: the probability of fixation/loss should be proportional to the global frequency of a morph [fixation ✗, loss ✓]

The stands we sampled were usually polymorphic for flower colour (Table 2; 89.4 % of 132 in 2021; 91.1 % of 112 in 2022; 88.8 % of 116 in 2023), and most of these polymorphic stands included all three colour morphs (73.7 % in 2023; 88.2 % in 2022; 93.2 % in 2023). Consistent with neutral expectations, monomorphic stands fixed for a single colour morph were about a tenth of the size of polymorphic stands and tended to be in areas with lower human activity (Table 3). In contrast to neutral expectations, monomorphic stands were almost always fixed for the purple morph. Fixed purple stands made up 86 % of 14 monomorphic stands in 2021, 90 % of 10 in 2022 and 92 % of 13 in 2023. This is significantly higher than would be expected based on the mean frequency of the purple morph in polymorphic stands (0.501 in 2021 χ^2^ = 7.10, simulated *P* = 0.019; 0.495 in 2022 χ^2^ = 6.56, *P* = 0.011; 0.428 in 2023 χ^2^ = 13.01, *P* = 0.00050). Moreover, all five stands that were monomorphic in all three years were fixed for purple (Table 2). When stands lacked one morph (23.5 % of stands in 2021, 10.7 % in 2022, 6.0 % in 2023), they usually lacked the white morph (Fig. 2; 93.5 % of 31 dimorphic stands in 2021, 83.3 % of 12 in 2022, 71.4 % of 7 in 2023). However, this did not differ from neutral expectations based on the relative low frequency of the white morph in polymorphic stands (Table 2; 0.144 in 2021 χ^2^ = 1.59, simulated *P* = 0.29; 0.190 in 2022 χ^2^ = 0.04, *P* > 0.9; 0.216 in 2023 χ^2^ = 0.20, *P* > 0.9).

**Table 2.** Variation in the frequency of colour morphs in stands of *Hersperis matronalis* from eastern Ontario, Canada, surveyed in three consecutive years (generations). Average morph frequency was calculated across all stands and from only stands polymorphic for flower colour. The bottom half of the table shows the numbers of stands fixed for each colour morph in each year and all years.

Subset	Year	*n* stands	Colour morph frequency		
			White	Pink	Purple
All stands	2021	132	0.129	0.333	0.538
	2022	112	0.174	0.295	0.531
	2023	116	0.192	0.324	0.484
Polymorphic stands	2021	118	0.144	0.355	0.501
	2022	102	0.190	0.314	0.495
	2023	103	0.216	0.356	0.428
			Number of stands fixed for:		
			White	Pink	Purple
Monomorphic stands	2021	14^1^	0	2	12
	2022	10^2^	0	1	9
	2023	13^3^	0	1	12
	All 3 years	5^4^	0	0	5

^1^Five of these stands were gone in 2022, two of which reappeared in 2023. Of the 11 resampled stands, four were polymorphic in future years, one just barely. Five stands had <5 plants but five had >30 plants. ^2^Six of these stands were also fixed in 2021 (five of which stayed fixed in 2023). Three were polymorphic in 2021. One was located in 2021 but only became accessible in 2022 and remained fixed in 2023. Three stands had <5 plants but four had >30 plants. ^3^Nine of these stands were fixed in a previous year (five for all three years). Four were polymorphic in a previous year (including two that were gone in 2022). Two stands had <5 plants but six had >30 plants. ^4^The mean size of these stands across all three years ranged from 19.3 to 144.0 and averaged 74.1.

**Table 3.** Comparison of stand size (log_10_*N*) and human activity (NSB) between stands of *Hesperis matronalis* monomorphic vs. polymorphic for flower colour. Monomorphic stands made up 14 of 132 stands in 2021, 10 of 112 stands in 2022 and 13 of 116 stands in 2023. Monomorphic and polymorphic stands were compared using Welch’s two-sample *t*-test with a one-tailed test of significance. Values in the monomorphic and polymorphic columns indicate mean ± s.e.

Variable	Year	Monomorphic	Polymorphic	*t*-test (one-tailed *P*)
Stand size log_10_*N*	2021	1.19 ± 0.17	2.11 ± 0.06	*t* = 5.08, *P* < 0.0001
	2022	1.29 ± 0.18	2.10 ± 0.06	*t* = 4.21, *P* = 0.0007
	2023	1.45 ± 0.21	2.26 ± 0.07	*t* = 3.63, *P* = 0.0012
Human activity NSB	2021	–21.5 ± 0.14	–20.9 ± 0.07	*t* = 3.73, *P* = 0.0006
	2022	–21.4 ± 0.26	–21.0 ± 0.08	*t* = 1.50, *P* = 0.080
	2023	–21.6 ± 0.07	–21.0 ± 0.08	*t* = 6.10, *P* < 0.0001

### Neutral prediction 4b: morph diversity should not be consistently shaped by the frequency of a particular morph [✗]

**Fig. 4.**
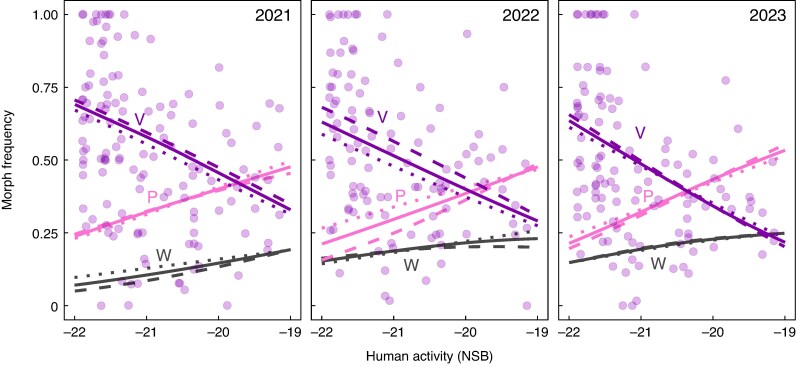
Systematic changes in flower colour morph frequencies with both human activity (NSB) and stand size (log_10_*N*) among stands of *Hesperis matronalis* in eastern Ontario, Canada, sampled over three years (132 stands in 2021, 112 in 2022, 116 in 2023). NSB and log_10_*N* interacted in their effects on morph frequencies (Table 3). Lines show the multinomial regression predictions for each colour morph (W = white, P = light pink, V = purple). Solid lines show predictions at the median log_10_*N*, dashed lines at the 25th percentile and dotted lines at the 75th percentile. Although the effect of log_10_*N* on the frequency of each morph varies with NSB, the effect of NSB is more consistent. The points show frequencies of the purple morph to emphasize the relatively weak predictive ability of these regressions (*r*^2^ values in Table 3).

In contrast to neutral expectations, multinomial logistic regression revealed that the frequencies of the three colour morphs covaried with both human activity (NSB) and stand size (log_10_*N*) and these effects interacted (Fig. 4; Table 4). In particular, the purple morph decreased in frequency with increasing human activity in all years and the slope of this relationship indicates that the purple morph was around 30 % more frequent in the most rural stands compared to most urban ones. This was associated with opposing but somewhat weaker and less consistent increases in the frequencies of the pink and white morphs. The effect of stand size (log_10_*N*) on morph frequencies was less consistent and depended on the level of human activity (Fig. 4). The increase in the frequency of the purple morph correlated negatively with morph diversity in all years (2021 *r* = −0.63, 2022 *r* = −0.68, 2023 *r* = −0.67, all *P* < 0.0001). To test whether human activity and stand size influenced morph diversity beyond just their influence on the frequency of the purple morph, we subsequently included colour morph diversity predicted from the systematic variation in morph frequencies (*H*′_pred_) as a covariate in the above linear models. There was some collinearity among predictors as expected because *H*′_pred_ correlated positively with both NSB (2021 *r* = +0.68, 2022 *r* = +0.63, 2023 *r* = +0.38, all *P* < 0.0001) and log_10_*N* (2021 *r* = +0.77, 2022 *r* = +0.65, 2023 *r* = +0.39, all *P* < 0.0001), but the VIF was <5 for all predictors in all years (2021: NSB = 2.12, log_10_*N* = 2.80, *H*′_pred_ = 4.53; 2022: NSB = 2.10, log_10_*N* = 2.16, *H*′_pred_ = 3.55; 2023: NSB = 1.17, log_10_*N* = 1.18, *H*′_pred_ = 1.36). Adding *H*′_pred_ weakened the effect of both NSB and log_10_*N* on *H*′ in 2023 (Table 1). However, *H*′_pred_ was not a significant predictor of *H*′ in 2021 or 2022.

**Table 4.** Analysis of variation in flower colour morph frequencies among stands of *Hesperis matronalis* in eastern Ontario, Canada. The numbers of the three colour morphs sampled in each stand were modelled as a multinomial response variable with human activity (NSB) and stand size (log_10_*N*) and their interaction as predictors. For all three years, the best model included the interaction. The bottom half of the table reports the strength of regressions of observed morph frequencies on morph frequencies predicted by the model.

Year	2021		2022		2023	
Multinomial regression						
Predictor	LRT χ^2^	*P*	LRT χ^2^	*P*	LRT χ^2^	*P*
Human activity (NSB)	373.3	<0.0001	374.1	<0.0001	567.2	<0.0001
Stand size (log_10_*N*)	73.5	<0.0001	154.8	<0.0001	10.5	0.0053
NSB × log_10_*N*	30.5	<0.0001	31.0	<0.0001	19.6	<0.0001
Observed vs. predicted morph frequencies						
	*r* ^2^	*P*	*r* ^2^	*P*	*r* ^2^	*P*
White	0.134	<0.0001	0.041	0.033	0.039	0.034
Pink	0.082	0.00083	0.271	<0.0001	0.169	<0.0001
Purple	0.177	<0.0001	0.230	<0.0001	0.173	<0.0001

### Neutral prediction 5: morphs should not differ in fitness [✓ mostly]

**Fig. 5.**
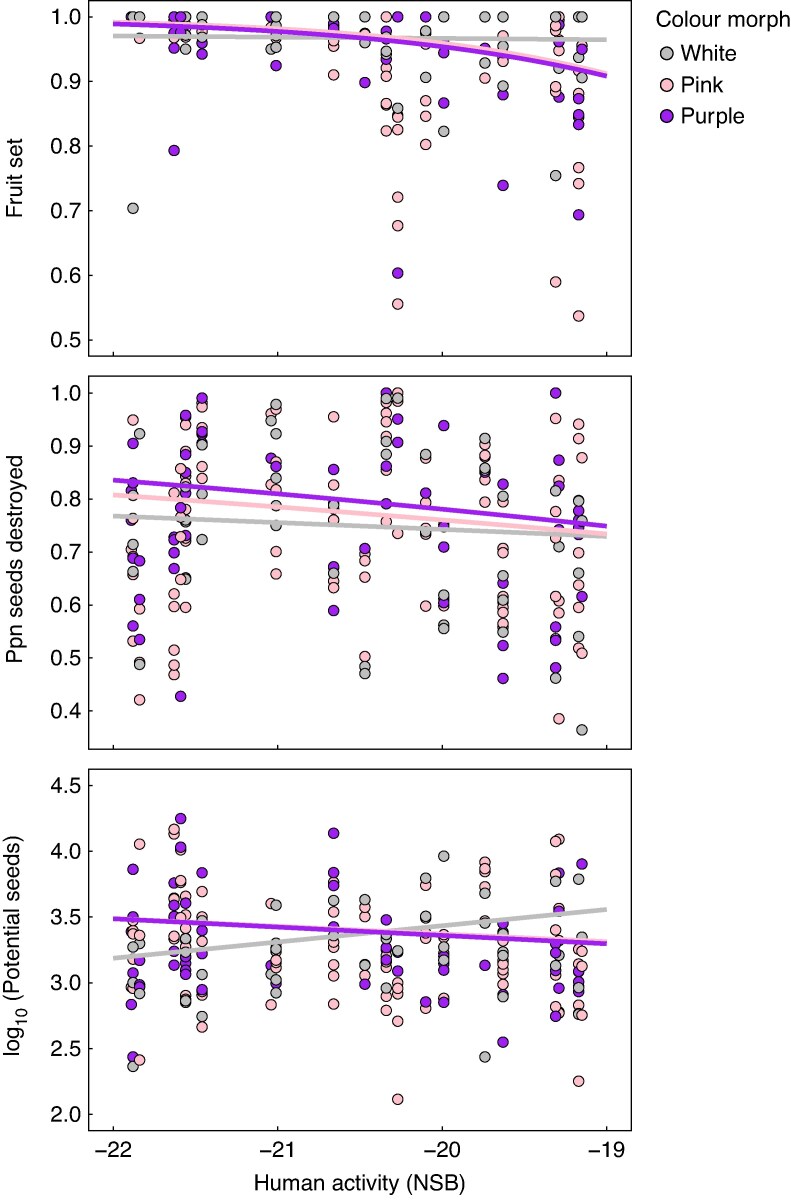
Variation in the proportion of flowers forming fruit (fruit set) and the proportion of seeds destroyed by the weevil seed predator *Ceutorhynchus inaffectatus* and the estimated potential seeds per plant in the absence of predation across a gradient of human activity and among flower colour morphs in stands of *Hesperis matronalis* from eastern Ontario, Canada. Points are individual plants from a subsample of 22 stands. Sample sizes were: white = 47 plants (grey points), pink = 109 (pink points), purple = 59 (purple points). Analysis of these data is shown in Table 5. Potential seeds per plant is log_10_-transformed for display only. The regression of fruit set and potential seeds over NSB varied significantly among morphs but mean fruit set or potential seeds did not vary among morphs. The regression of proportion seeds destroyed over NSB also varied among morphs and was, on average, higher for the purple morph, lower for the pink morph and lower still for the white morph. No other fitness measures varied with NSB or among morphs.

The plants we sampled in 2021 varied dramatically in size and reproductive success. Dry stem mass varied 100-fold among individuals (0.68–72.65 g) with a median of 5.57 g. Flowers produced per individual varied more than 50-fold (11–736 flowers, median = 67). The number of filled seeds per plant ranged from 0 to 10 146 (median = 378). Despite this wide variation, none of these measures differed among colour morphs (Table 5). The proportion of flowers forming fruit (fruit set) declined slightly with increasing human activity for the pink and purple morphs but not the white morph (Fig. 5). However, fruit set was generally high (mean = 0.95, fruit set = 1 for 48 % of plants) and did not vary among morphs on average. Much of the variation in seed production among plants was due to pre-dispersal seed predation by the adventive weevil *C. inaffectatus*. Predation was near ubiquitous as all plants had at least one of five sampled fruits damaged, and 73.0 % of plants suffered damage to all five fruits. All individuals had some seeds destroyed, and the proportion of seeds destroyed varied from 0.36 to 1.00 (median = 0.76). Although this seed predator was also introduced from Europe and thus could be potentially more prevalent in areas with higher human activity, the proportion of seeds destroyed generally decreased with increasing human activity. The decrease was steeper for the purple and pink morphs than the white morph, and contrary to predictions, mean seed predation was highest in the purple morph (∼6 % higher than among white morph plants) and intermediate in the pink morph. Although variation in seed predation among morphs was not reflected in differences in per-capita realized seed production among morphs, estimated potential seed production per plant in the absence of predation increased by 135 % with increasing urbanization for the white morph and decreased by 36 % for the pink and purple morphs. As a result, the pink and purple morphs had a higher potential for seed production than the white morph in rural habitat and lower in urban habitat (Fig. 5).

**Table 5.** Analysis of variation in plant size, flower, fruit and seed production and seed predation among flower colour morphs of *Hersperis matronalis* across 22 sites along a gradient of human activity (NSB) in eastern Ontario, Canada (these 22 sites are a subsample of the 132 sites sampled in 2021). Variation in each response variable was fit to a generalized linear model with colour morph (df = 2), NSB (df = 1) and their interaction (df = 2) as fixed effects, site as a random effect, and an error distribution appropriate for the response variable (Errors, Neg bin = negative binomial). We evaluated significance of colour morph using chi-squared likelihood ratio tests (LRT). Data for fruit set (proportion of flowers forming fruit) and proportion seeds destroyed are displayed in Fig. 5.

		LRT χ^2^ (*P*)		
Response variable	Errors	Colour morph (M)	NSB	M × NSB
log_10_(stem mass, g)	Gaussian	0.59 (0.74)	0.70 (0.40)	2.98 (0.22)
Flowers per plant	Neg bin	0.08 (0.96)	0.12 (0.73)	3.07 (0.21)
Fruit set	Binomial	4.59 (0.10)	9.62 (0.0019)	34.91 (<0.0001)
Fruits per plant	Neg bin	0.03 (0.99)	0.28 (0.59)	3.98 (0.14)
Seeds per plant	Neg bin	0.52 (0.77)	0.15 (0.70)	2.85 (0.24)
Potential seeds per plant	Neg bin	0.01 (0.99)	0.30 (0.58)	8.57 (0.014)
Proportion seed destroyed	Binomial	36.45 (<0.0001)	0.64 (0.42)	6.65 (0.036)

## DISCUSSION

We investigated the evolutionary forces influencing flower colour variation among 136 stands of an introduced biennial plant along an urban–rural gradient in eastern Ontario, Canada, over three consecutive generations. Taken together, our results are consistent with colour morph frequencies being influenced by the interplay of non-selective evolutionary processes such as genetic drift and gene flow. In line with neutral predictions 1 and 2, colour morph diversity was higher in larger stands and in areas of higher human activity where horticultural introductions and the dispersal of seeds are probably more frequent. Moreover, among-generation variation in colour diversity was lower in stands that were (1) larger, (2) less variable in year-to-year stand size and (3) in more urban environments, though the effect of human activity was not significant (neutral prediction 3). However, in contrast to neutral prediction 4, we detected non-random variation in morph frequencies. The purple morph was more common in smaller stands and in rural areas, and this was associated with the fixation of the purple morph in some rural stands. Accounting for the non-random trends in morph frequencies only slightly (and usually non-significantly) reduced the associations between colour morph diversity and both stand size and human activity. Colour morphs did not differ in above-ground size, flower production or lifetime seed production (neutral prediction 5). Unexpectedly, plants with purple flowers suffered the most damage by seed predators and this damage decreased with increasing human activity. However, variation in seed predation was not reflected in differences among morphs in total seed production.

In agreement with neutral expectations, flower colour morph diversity correlated positively with stand size in all three generations. Similarly, the between-generation variation in *H*′ was lower in stands that maintained a larger mean size or exhibited less variation in size across generations. Because flower colour morph diversity probably represents genetic variation at the loci responsible for flower colour, this is consistent with the expectation that genetic diversity is higher in larger populations, which has been supported by analyses of putatively neutral protein and DNA sequence variation in diverse species, especially plants (Frankham, 1996; Leimu and Fischer, 2008). Population size also affects genetic variation underlying morphological polymorphisms that may potentially influence fitness, for instance style morph frequencies in heterostylous plants where morph frequencies are under negative frequency-dependent selection (Eckert *et al*., 1996; Costa *et al*., 2016). Similarly, population size also correlated positively with morphological variation in *Salvia pratensis* and *Scabiosa columbaria* (Ouborg *et al*., 1991). Although analysis of variation in flower colour morph frequencies, specifically in *Linanthus parryae*, was involved in some of the first tests of neutral processes in nature (Epling and Dobzhansky, 1942; Schemske and Bierzychudek, 2001), few studies have since investigated the relation between population size and flower colour morph diversity, within populations, across space or time (Sapir *et al*., 2021). In *Cirsium palustre*, morph frequencies did not consistently vary with population size, but increased population size from year to year was associated with increased flower colour morph diversity (Mogford, 1974). A small number of studies have looked at the relation between population size and the composition of flower colour morph frequencies within populations, without considering morph diversity (Jersáková *et al*., 2006; Imbert, 2021).

Habitat fragmentation associated with urbanization often reduces population size and gene flow, thereby making urban populations more vulnerable to the loss of genetic variation via genetic drift (Bartlewicz *et al*., 2015; Munshi-South *et al*., 2016; Miles *et al*., 2019). In contrast, we predicted larger stand size and higher colour morph diversity in urban areas. This is because the introduction and spread of non-native species, particularly plants used in horticulture, are often associated with human activity (Barney, 2006; Pairon *et al*., 2010). Among the stands of *H. matronalis* we sampled, log-transformed stand size correlated positively, though not strongly, with human activity (measured as NSB, *r* ≤ +0.35, see Methods), and human activity positively influenced colour morph diversity independently of variation in stand size. *Hesperis matronalis* was introduced into North America as a garden ornamental more than 200 years ago and continues to be sold for that purpose (Francis *et al*., 2009). It is likely that there have been multiple independent introductions of *H. matronalis* from gardens into naturalized populations. We searched the internet and found 26 North American seed retailers that currently sell *H. matronalis* (one of the 26 retail sites provided no information on flower colour and is not included in the following discussion). Of these retailers, 48 % advertised their seed stock as including more than one flower colour (Supplementary Data Appendix S8). There are more gardens in urban areas, and individual gardens may be planted with different seed stock (which may have different starting ratios of colour morphs). Simply by having more independent introduction, gardens in urban areas may contribute to higher diversity in escaped naturalized stands, since individual garden varieties can later mix in naturalized stands. As a result, urban gardens may serve as a source of flower colour diversity for naturalized populations, like the ones we sampled.

An alternative explanation, not mutually exclusive of multiple introductions, is that urban populations of this non-native species are in closer proximity to one another than those in rural populations, which would enhance the maintenance of genetic variation at flower colour loci via gene flow in urban areas. Field observations made during three years of sampling *H. matronalis* in eastern Ontario clearly indicated that stands of *H. matronalis* are much more frequent and closer together in urban than rural areas, as has been observed with other introduced species (Johnson *et al*., 2018). However, variation in the proximity of natural populations is near impossible to formally quantify for common plant species in complex landscapes, and *H. matronalis* is no exception. Particularly in urban areas, our sampled stands represent only a fraction of the existing naturalized stands. Therefore, the distance between stands in this study do not represent the true isolation of stands. Records of the species on iNaturalist (inaturalist.ca) are clearly more frequent closer to Kingston city centre (especially if the records from our systematic survey are excluded), but this is probably due to a general trend of more iNaturalist posts and contributors closer to urban areas (Di Cecco *et al*., 2021).

Contrary to neutral expectations, we observed that morph frequencies had consistent associations with human activity. The purple morph increased in frequency in areas with lower human activity and was fixed in more rural stands than would be expected by chance. If flower colour was a neutral trait, theory would predict that the probability of any colour morph becoming fixed is equal to the frequency of each morph in the global population (Hedrick, 2000). In contrast, more than 80 % of fixed stands were fixed for the purple morph despite the frequency of the purple morph being 40–50 % depending on the study year. It is possible that the observed spatial pattern in morph frequency is simply the result of historical contingency (Eckert and Barrett, 1995; Koski and Galloway, 2020). Of the retailers that sell *H. matronalis* seeds, 52 % appear to offer seed stock that is monomorphic for flower colour, often purple (Supplementary Data Appendix S8). It is possible that there was an early introduction of purple morph seeds to the rural end of our urbanization gradient. However, if the dominance of the purple morph in rural areas is due to happenstance, we might expect to see spatial clustering of stands with a high proportion of purple flowers around the site of the historical accident. While we did find that four stands monomorphic for purple in 2021 formed a spatial cluster, the majority did not (see Appendix S7 for a consideration of spatial autocorrelation). Historical contingency cannot be ruled out, but it is not a compelling explanation for non-random variation in morph frequencies.

Most studies of flower colour clines have focused on geographical variation in selective pressures, particularly pollinator interactions (Sapir *et al*., 2021). Of course, other biotic and abiotic factors, such as herbivores and climate, can also exert selection on patterns of flower colour variation (Strauss and Whittall, 2007; Caruso *et al*., 2019; Koski and Galloway, 2020). However, our analysis of variation in plant size, seed predation and seed production among morphs (albeit with modest and unbalanced samples) suggests that the dominance of the purple morph in rural stands of *H. matronalis* is not easily explained by differential selection of the colour morphs along the urban–rural gradient. Most measures of plant performance did not vary among colour morphs or with human activity. We did find that the fruit set of purple and pink morphs, but not white, decreased with increasing human activity and that the colour morphs differed in pre-dispersal seed predation, particularly at rural sites. However, these effects were not reflected in differences among morphs in lifetime seed production (see also Mitchell and Ankeny, 2001; Weeks and Frey, 2007).

An important caveat for our comparison of fitness correlates among morphs is that we have only measured lifetime seed fitness. Thus, it remains possible that there is a significant difference between morphs in some other aspects of fitness such as early survival or pollen-related components. Asymmetries in pollen-related fitness could arise through either differences in pollen production or pollinator visitation, which is especially relevant since *H. matronalis* is probably predominantly outcrossing (Mitchell and Ankeny, 2001; Weeks and Frey, 2007; but see Susko and Clubb, 2008). For instance, Mitchell and Ankeny (2001) found that, in three *H. matronalis* populations in Ohio, ‘pink’ flowers had 6 % larger petals than ‘pale’ flowers, and pollinators preferentially visited plants with larger flowers (Weeks and Frey, 2007). In contrast, Majetic *et al*. (2008) did not find any variation in pollinator visitation among *H. matronalis* colour morphs in their experimental arrays. To explain the systematic variation in morph frequencies we observed, differences among morphs in pollen fitness would have to vary across the urban–rural gradient, potentially via changes in the composition of pollinator communities or co-flowering species.

Floral pigments can affect plant fitness in a variety of ways, including resistance to herbivory (Simms and Bucher, 1996; Fineblum and Rausher, 1997; Johnson *et al*., 2015). For example, anthocyanin, the pigment involved in flower colour variation in *H. matronalis* (Majetic *et al.*, 2008), reduces herbivory in several species (Irwin *et al*., 2003; Strauss *et al*., 2004; Vaidya *et al*., 2018). Much of the variation in seed production among the plants we sampled was due to seed predation by the adventive weevil *C. inaffectatus* and we predicted that the purple morph by virtue of higher anthocyanin concentrations would experience lower seed predation. Because this specialist seed predator was first observed in Ontario only a few years before our study (2018, Pentinsaari *et al*., 2019), we also expected it would be most prevalent in areas with higher human activity. Neither of these expectations were supported by our results. Seed predation was highest on the purple morph and declined with increasing urbanization. This result is not unprecedented. In *Claytonia virginica*, a species with a pink and white flower colour polymorphism, plants with more pigmented flowers have lower levels of defensive flavanols and receive more damage from herbivorous slugs (Harborne, 1979; Frey, 2004). Additionally, some specialist insect herbivores can be unaffected and even attracted to the defensive compounds of their host (Giamoustaris and Mithen, 1995; Lankau, 2007; but see Agrawal and Kurashige, 2003). Future research should more directly investigate the relation between floral pigmentation and the concentration of defensive secondary metabolites in *H. matronalis*.

The relatively recent arrival of *C. inaffectatus* may have obscured past fitness differences between morphs that are still reflected in the urban–rural cline in colour morph frequency variation that we observed. Our results show that seed predation was highest on the purple morph, especially at the rural end of the gradient. Hence the recent arrival of this seed predator could erase a previous seed fitness advantage of the purple morph in rural populations which could have explained the increasing dominance of the purple morph in rural areas. This is partially supported by our analysis of estimated seed production per plant in the absence of predation. Relative potential seed production of the purple and pink morphs (when the effect of predation was removed) was higher than that of the white morph in rural areas and lower than that of the white morph in urban areas. This ‘ghost of fitness differences past’ hypothesis could be tested by manipulating *C. inaffectatus* seed predation by applying insecticides and by comparing our results to surveys of morph frequencies along replicate urban–rural gradients in regions where *C. inaffectatus* has yet to invade.

## Supplementary Material

mcag035_Supplementary_Data

## Data Availability

Data will be archived at DRYAD (https://datadryad.org/)

## Supplementary Data

Appendix S1: Locations of study sites and variation in human activity. Appendix S2: Spectrometric analysis of flower colour variation. Appendix S3: Analysis of colour morph diversity based on two morphs. Appendix S4: Combined analysis of data from 2021, 2022 and 2023. Appendix S5: Analysis of the between-year change in colour morph diversity. Appendix S6: Variation in colour morph diversity among generations. Appendix S7: Distribution of stands with a high frequency of the purple morph and a spatial autocorrelation analysis. Appendix S8: Retailers of *Hesperis matronalis* seed in North America.

## References

[mcag035-B1] Agrawal AA, Kurashige NS. 2003. A role for isothiocyanates in plant resistance against the specialist herbivore *Pieris rapae*. Journal of Chemical Ecology 29: 1403–1415. doi:10.1023/A:1024265420375 12918924

[mcag035-B2] Alberti M, Palkovacs EP, Des Roches S, et al 2020. The complexity of urban eco-evolutionary dynamics. BioScience 70: 772–793. doi:10.1093/biosci/biaa079

[mcag035-B3] Austerlitz F, Jung-Muller B, Godelle B, Gouyon PH. 1997. Evolution of coalescence times, genetic diversity and structure during colonization. Theoretical Population Biology 51: 148–164. doi:10.1006/tpbi.1997.1302

[mcag035-B4] Barney JN . 2006. North American history of two invasive plant species: phytogeographic distribution, dispersal vectors, and multiple introductions. Biological Invasions 8: 703–717. doi:10.1007/s10530-005-3174-9

[mcag035-B5] Barrett SCH . 2019. A most complex marriage arrangement: recent advances on heterostyly and unresolved questions. New Phytology 224: 1051–1067. doi:10.1111/nph.16026 31631362

[mcag035-B6] Barrett SCH, Morgan MT, Husband BC. 1989. The dissolution of a complex genetic polymorphism: the evolution of self-fertilization in tristylous *Eichhornia paniculata* (Pontederiaceae). Evolution 43: 1398–1416. doi:10.1111/j.1558-5646.1989.tb02591.x 28564241

[mcag035-B7] Bartlewicz J, Vandepitte K, Jacquemyn H, Honnay O. 2015. Population genetic diversity of the clonal self-incompatible herbaceous plant *Linaria vulgaris* along an urbanization gradient. Biological Journal of the Linnean Society 116: 603–613. doi:10.1111/bij.12602

[mcag035-B8] Cadotte MW, Yasui SLE, Livingstone S, MacIvor JS. 2017. Are urban systems beneficial, detrimental, or indifferent for biological invasion? Biological Invasions 19: 3489–3503. doi:10.1007/s10530-017-1586-y

[mcag035-B9] Caruso CM, Eisen KE, Martin RA, Sletvold N. 2019. A meta-analysis of the agents of selection on floral traits. Evolution 73: 4–14. doi:10.1111/evo.13639 30411337

[mcag035-B10] Costa J, Castro S, Loureiro J, Barrett SCH. 2016. Variation in style morph frequencies in tristylous *Lythrum salicaria* in the Iberian Peninsula: the role of geographical and demographic factors. Annals of Botany 117: 331–340. doi:10.1093/aob/mcv173 26658100 PMC4724046

[mcag035-B11] Davis AR, Pylatuik JD, Paradis JC, Low NH. 1998. Nectar-carbohydrate production and composition vary in relation to nectary anatomy and location within individual flowers of several species of Brassicaceae. Planta 205: 305–318. doi:10.1007/s004250050325 9637073

[mcag035-B12] Dehnen-Schmutz K, Touza J, Perrings C, Williamson M. 2007. The horticultural trade and ornamental plant invasions in Britain. Conservation Biology 21: 224–231. doi:10.1111/j.1523-1739.2006.00538.x 17298528

[mcag035-B13] Di Cecco GJ, Barve V, Belitz MW, Stucky BJ, Guralnick RP, Hurlbert AH. 2021. Observing the observers: how participants contribute data to iNaturalist and implications for biodiversity science. BioScience 71: 1179–1188. doi:10.1093/biosci/biab093

[mcag035-B14] Dlugosch KM, Parker IM. 2008. Founding events in species invasions: genetic variation, adaptive evolution, and the role of multiple introductions. Molecular Ecology 17: 431–449. doi:10.1111/j.1365-294X.2007.03538.x 17908213

[mcag035-B15] Eckert CG, Barrett SCH. 1995. Style morph ratios in tristylous *Decodon verticillatus* (Lythraceae): selection vs. historical contingency. Ecology 76: 1051–1066. doi:10.2307/1940915

[mcag035-B16] Eckert CG, Manicacci D, Barrett SCH. 1996. Frequency-dependent selection on morph ratios in tristylous *Lythrum salicaria* (Lythraceae). Heredity 77: 581–588. doi:10.1038/hdy.1996.185

[mcag035-B17] Epling C, Dobzhansky T. 1942. Genetics of natural populations. VI. microgeographic races in *Linanthus parryae*. Genetics 27: 317–332. doi:10.1093/genetics/27.3.317 17247043 PMC1209161

[mcag035-B18] Faegri K, van der Pijl L. 1979. Principles of pollination ecology, 3rd edn. New York: Pergamon Press.

[mcag035-B19] Falchi F, Cinzano P, Duriscoe D, et al 2016. The new world atlas of artificial night sky brightness. Science Advances 2: e1600377. doi:10.1126/sciadv.1600377 27386582 PMC4928945

[mcag035-B20] Finand B, Loeuille N, Bocquet C, Fédérici P, Ledamoisel J, Monnin T. 2024. Habitat fragmentation through urbanization selects for low dispersal in an ant species. Oikos 2024: e10325. doi:10.1111/oik.10325

[mcag035-B21] Fineblum WL, Rausher MD. 1997. Do floral pigmentation genes also influence resistance to enemies? The W locus in *Ipomoea purpurea*. Ecology 78: 1646–1654. doi:10.1890/0012-9658(1997)078[1646:DFPGAI]2.0.CO;2

[mcag035-B22] Francis A, Cavers PB, Warwick SI. 2009. The biology of Canadian weeds. 140. *Hesperis matronalis* L. Canadian Journal of Plant Science 89: 191–206. doi:10.4141/CJPS08094

[mcag035-B23] Frankham R . 1996. Relationship of genetic variation to population size in wildlife. Conservation Biology 10: 1500–1508. doi:10.1046/j.1523-1739.1996.10061500.x

[mcag035-B24] Frey FM . 2004. Opposing natural selection from herbivores and pathogens may maintain floral-color variation in *Claytonia virginica* (Portulacaceae). Evolution 58: 2426–2437. doi:10.1111/j.0014-3820.2004.tb00872.x 15612286

[mcag035-B25] Giamoustaris A, Mithen R. 1995. The effect of modifying the glucosinolate content of leaves of oilseed rape (*Brassica napus* ssp. oleifera) on its interaction with specialist and generalist pests. The Annals of Applied Biology 126: 347–363. doi:10.1111/j.1744-7348.1995.tb05371.x

[mcag035-B26] Harborne JB . 1979. Flavinoid pigments. In: Rosenthal GA, Janzen DH. eds. Herbivores, their interaction with secondary plant metabolites. New York: Academic Press, 619–655.

[mcag035-B27] Harris C, Jiang H, Liu D, Brian Z, He K. 2009. Testing the roles of species native origin and family membership in intentional plant introductions using nursery data across the state of Kentucky. Journal of the Torrey Botanical Society 136: 122–127. doi:10.3159/08-RA-080R1.1

[mcag035-B28] Hedrick PW . 2000. Genetics of populations, 2nd edn. London: Jones and Bartlett.

[mcag035-B29] Imbert E . 2021. Spatial distribution of flower color polymorphism in *Iris lutescens*. Botany Letters 168: 408–421. doi:10.1080/23818107.2020.1833750

[mcag035-B30] Irwin RE, Strauss SY, Storz S, Emerson A, Guibert G. 2003. The role of herbivores in the maintenance of flower color polymorphism in wild radish. Ecology 84: 1733–1743. doi:10.1890/0012-9658(2003)084[1733:TROHIT]2.0.CO;2

[mcag035-B31] Jersáková J, Kindlmann P, Renner SS. 2006. Is the color dimorphism in *Dactylorhiza sambucina* maintained by differential seed viability instead of frequency-dependent selection? Folia Geobotanica 41: 61–76. doi:10.1007/BF02805262

[mcag035-B32] Johnson MTJ, Campbell SA, Barrett SCH. 2015. Evolutionary interactions between plant reproduction and defense against herbivores. Annual Review of Ecology, Evolution, and Systematics 46: 191–213. doi:10.1146/annurev-ecolsys-112414-054215

[mcag035-B33] Johnson MTJ, Munshi-South J. 2017. Evolution of life in urban environments. Science 358: eaam8327. doi:10.1126/science.aam8327 29097520

[mcag035-B34] Johnson MTJ, Prashad CM, Lavoignat M, Saini HS. 2018. Contrasting the effects of natural selection, genetic drift and gene flow on urban evolution in white clover (*Trifolium repens*). Proceedings of the Royal Society B 285: 20181019. doi:10.1098/rspb.2018.1019 30051843 PMC6083247

[mcag035-B35] Joron M, Papa R, Beltrán M, et al 2006. A conserved supergene locus controls colour pattern diversity in *Heliconius* butterflies. PLoS Biology 4: e303. doi:10.1371/journal.pbio.0040303 17002517 PMC1570757

[mcag035-B36] Karageorgou P, Manetas Y. 2006. The importance of being red when young: anthocyanins and the protection of young leaves of *Quercus coccifera* from insect herbivory and excess light. Tree Physiology 26: 613–621. doi:10.1093/treephys/26.5.613 16452075

[mcag035-B37] Kingsley EP, Manceau M, Wiley CD, Hoekstra HE. 2009. Melanism in *Peromyscus* is caused by independent mutations in agouti. PLoS One 4: e6435. doi:10.1371/journal.pone.0006435 19649329 PMC2713407

[mcag035-B38] Koski MH, Galloway LF. 2020. Geographic variation in floral color and reflectance correlates with temperature and colonization history. Frontiers in Plant Science 11: 991. doi:10.3389/fpls.2020.00991 32714360 PMC7340105

[mcag035-B39] Kowarik I . 2003. Human agency in biological invasions: secondary releases foster naturalisation and population expansion of alien plant species. Biological Invasions 5: 293–312. doi:10.1023/B:BINV.0000005574.15074.66

[mcag035-B40] Lankau RA . 2007. Specialist and generalist herbivores exert opposing selection on a chemical defense. New Phytologist 175: 176–184. doi:10.1111/j.1469-8137.2007.02090.x 17547677

[mcag035-B41] Larsen LM, Nielsen JK, Sorensen H. 1992. Host plant recognition in monophagous weevils: specialization of *Ceutorhynchus inaffectatus* to glucosinolates from its host plant *Hesperis matronalis*. Entomologia Experimentalis et Applicata 64: 49–55. doi:10.1111/j.1570-7458.1992.tb01593.x

[mcag035-B42] Leimu R, Fischer M. 2008. A meta-analysis of local adaptation in plants. PLoS One 3: e4010. doi:10.1371/journal.pone.0004010 19104660 PMC2602971

[mcag035-B43] Lev-Yadun S, Gould KS. 2008. Role of anthocyanins in plant defence. In: Winefield C, Davies K, Gould K. eds. Anthocyanins. New York: Springer, 22–28.

[mcag035-B44] Lu S, Luo X, Han L, Yang J, Jin J, Yang J. 2022. Genetic patterns reveal differences between the invasion processes of common ragweed in urban and non-urban ecosystems. Global Ecology and Conservation 38: e02214. doi:10.1016/j.gecco.2022.e02214

[mcag035-B45] Mack RN, Erneberg M. 2002. The United States naturalized flora: largely the product of deliberate introductions. Annals of the Missouri Botanical Garden 89: 176–189. doi:10.2307/3298562

[mcag035-B46] Mairal M, Chown SL, Shaw J, et al 2022. Human activity strongly influences genetic dynamics of the most widespread invasive plant in the sub-Antarctic. Molecular Ecology 31: 1649–1665. doi:10.1111/mec.16045 34181792

[mcag035-B47] Majetic CJ, Raguso RA, Ashman TL. 2008. The impact of biochemistry vs. population membership on floral scent profiles in color polymorphic *Hesperis matronalis*. Annals of Botany 102: 911–922. doi:10.1093/aob/mcn181 18819948 PMC2712399

[mcag035-B48] Majetic Cassie J., Raguso Robert A., Tonsor Stephen J., Ashman Tia-Lynn. 2007. Flower color–flower scent associations in polymorphic Hesperis matronalis (Brassicaceae). Phytochemistry 68: 865–874. 10.1016/j.phytochem.2006.12.009 17258250

[mcag035-B49] McLean P, Gallien L, Wilson JRU, Gaertner M, Richardson DM. 2017. Small urban centres as launching sites for plant invasions in natural areas: insights from South Africa. Biological Invasions 19: 3541–3555. doi:10.1007/s10530-017-1600-4

[mcag035-B50] Meléndez-Ackerman E, Campbell DR. 1998. Adaptive significance of flower color and inter-trait correlations in an *Ipomopsis* hybrid zone. Evolution 52: 1293–1303. doi:10.1111/j.1558-5646.1998.tb02011.x 28565388

[mcag035-B51] Miles LS, Rivkin RL, Johnson MTJ, Munshi-South J, Verrelli BC. 2019. Gene flow and genetic drift in urban environments. Molecular Ecology 28: 4138–4151. doi:10.1111/mec.15221 31482608

[mcag035-B52] Mitchell RJ, Ankeny DP. 2001. Effects of local conspecific density on reproductive success in *Penstemon digitalis* and *Hesperis matronalis*. Ohio Journal of Science 101: 22–27. doi: 1811/23885

[mcag035-B53] Mogford DJ . 1974. Flower color polymorphism in *Cirsium palustre*. Heredity 33: 257–263. doi:10.1038/hdy.1974.90

[mcag035-B54] Morris EK, Caruso T, Buscot François M, et al 2014. Choosing and using diversity indices: insights for ecological applications from the German Biodiversity Exploratories. Ecology and Evolution 4: 3514–3524. doi:10.1002/ece3.1155 25478144 PMC4224527

[mcag035-B55] Munshi-South J, Zolnik CP, Harris SE. 2016. Population genomics of the Anthropocene: urbanization is negatively associated with genome-wide variation in white-footed mouse populations. Evolutionary Applications 9: 546–564. doi:10.1111/eva.12357 27099621 PMC4831458

[mcag035-B56] Naing AH, Kim CK. 2021. Abiotic stress-induced anthocyanins in plants: their role in tolerance to abiotic stresses. Physiologia Plantarum 172: 1711–1723. doi:10.1111/ppl.13373 33605458

[mcag035-B57] Noël S, Lapointe FJ. 2010. Urban conservation genetics: study of a terrestrial salamander in the city. Biological Conservation 143: 2823–2831. doi:10.1016/j.biocon.2010.07.033

[mcag035-B58] Ouborg NJ, van Treuren R, van Damme JMM. 1991. The significance of genetic erosion in the process of extinction: morphological variation and fitness components in populations of varying size of *Salvia pratensis* L. and *Scabiosa columbaria* L. Oecologia 86: 359–367. doi:10.1007/BF00317601 28312921

[mcag035-B59] Pairon M, Petitpierre B, Campbell M, et al 2010. Multiple introductions boosted genetic diversity in the invasive range of black cherry (*Prunus serotina*; Rosaceae). Annals of Botany 105: 881–890. doi:10.1093/aob/mcq065 20400456 PMC2876008

[mcag035-B60] Pentinsaari M, Anderson R, Borowiec L, et al 2019. DNA barcodes reveal 63 overlooked species of Canadian beetles (Insecta, Coleoptera). ZooKeys 894: 53–150. doi:10.3897/zookeys.894.37862 31844409 PMC6906170

[mcag035-B61] Pun CSJ, So CW. 2012. Night-sky brightness monitoring in Hong Kong: a city-wide light pollution assessment. Environmental Monitoring and Assessment 184: 2537–2557. doi:10.1007/s10661-011-2136-1 21713499

[mcag035-B62] Rausher MD . 2008. Evolutionary transitions in floral color. International Journal of Plant Sciences 169: 7–21. doi:10.1086/523358

[mcag035-B63] R Core Team . 2022. R: A Language and Environment for Statistical Computing. R Foundation for Statistical Computing, Vienna. https://www.R-project.org.

[mcag035-B64] Reichard SH, White P. 2001. Horticulture as a pathway of invasive plant introductions in the United States. BioScience 51: 103–113. doi:10.1641/0006-3568(2001)051[0103:HAAPOI]2.0.CO;2

[mcag035-B65] Rivkin RL, Johnson MTJ. 2022. The impact of urbanization on outcrossing rate and population genetic variation in the native wildflower, *Impatiens capensis*. Journal of Urban Ecology 8: juac009. doi:10.1093/jue/juac009

[mcag035-B66] Roman J, Darling JA. 2007. Paradox lost: genetic diversity and the success of aquatic invasions. Trends in Ecology and Evolution 22: 454–464. doi:10.1016/j.tree.2007.07.002 17673331

[mcag035-B67] Rothfels CJ, Beaton LL, Dudley SA. 2002. The effects of salt, manganese, and density on life history traits in *Hesperis matronalis* L. from old field and roadside populations. Canadian Journal of Botany 80: 131–139. doi:10.1139/b01-142

[mcag035-B68] Rousseau C . 1968. Histoire, habitat et distribution de 220 plantes introduites au Québec. Naturaliste Canadien 95: 49–171.

[mcag035-B69] Santangelo JS, Johnson MTJ, Ness RW. 2018. Modern spandrels: the roles of genetic drift, gene flow and natural selection in the evolution of parallel clines. Proceedings of the Royal Society London B 285: 20180230. doi:10.1098/rspb.2018.0230 PMC5966599 29743253

[mcag035-B70] Santangelo JS, Ness RW, Cohan B, et al 2022. Global urban environmental change drives adaptation in white clover. Science 375: 1275–1281. doi:10.1126/science.abk0989 35298255

[mcag035-B71] Sapir Y, Gallagher MK, Senden E. 2021. What maintains flower color variation within populations? Trends in Ecology and Evolution 36: 507–519. doi:10.1016/j.tree.2021.01.011 33663870

[mcag035-B72] Schemske DW, Bierzychudek P. 2001. Evolution of flower color in the desert annual *Linanthus parryae*: Wright revisited. Evolution 55: 1269–1282. doi:10.1111/j.0014-3820.2001.tb00650.x 11525452

[mcag035-B73] Schemske DW, Bierzychudek P. 2007. Spatial differentiation for flower color in the desert annual *Linanthus parryae*: was Wright right? Evolution 61: 2528–2543. doi:10.1111/j.1558-5646.2007.00219.x 17894812

[mcag035-B74] Simms EL, Bucher MA. 1996. Pleiotropic effects of flower-color intensity on herbivore performance on *Ipomoea purpurea*. Evolution 50: 957–963. doi:10.1111/j.1558-5646.1996.tb03908.x 28568918

[mcag035-B75] Slatkin M, Excoffier L. 2012. Serial founder effects during range expansion: a spatial analog of genetic drift. Genetics 191: 171–181. doi:10.1534/genetics.112.139022 22367031 PMC3338258

[mcag035-B76] Strauss SY, Irwin RE, Lambrix VM. 2004. Optimal defence theory and flower petal color predict variation in the secondary chemistry of wild radish. The Journal of Ecology 92: 132–141. doi:10.1111/j.1365-2745.2004.00843.x

[mcag035-B77] Strauss SY, Whittall JB. 2007. Non-pollinator agents of selection on floral traits. In: Harder LD, Barrett SCH. eds. Ecology and evolution of flowers. Oxford: Oxford University Press, 120–138.

[mcag035-B78] Susko DJ, Clubb M. 2008. Pollination effects on patterns of ovule fate in *Hesperis matronalis* (Brassicaceae). Botany 86: 466–474. doi:10.1139/B08-017

[mcag035-B79] Sutton P, Roberts D, Elvidge C, Baugh K. 2001. Census from Heaven: an estimate of the global human population using night-time satellite imagery. International Journal of Remote Sensing 22: 3061–3076. doi:10.1080/01431160010007015

[mcag035-B80] Thompson KA, Renaudin M, Johnson MTJ. 2016. Urbanization drives the evolution of parallel clines in plant populations. Proceedings of the Royal Society London B 283: 20162180. doi:10.1098/rspb.2016.2180 PMC5204167 28003451

[mcag035-B81] Vaidya P, McDurmon A, Mattoon E, et al 2018. Ecological causes and consequences of flower color polymorphism in a self-pollinating plant (*Boechera stricta*). New Phytologist 218: 380–392. doi:10.1111/nph.14998 29369384

[mcag035-B82] van Kleunen M, Essl F, Pergl J, et al 2018. The changing role of ornamental horticulture in alien plant invasions. Biological Reviews of the Cambridge Philosophical Society 93: 1421–1437. doi:10.1111/brv.12402 29504240

[mcag035-B83] Vicente S, Máguas C, Richardson DM, Trindade H, Wilson JRU, Le Roux JJ. 2021. Highly diverse and highly successful: invasive Australian acacias have not experienced genetic bottlenecks globally. Annals of Botany 128: 149–157. doi:10.1093/aob/mcab053 33876193 PMC8324033

[mcag035-B84] Waser NM, Price MV. 1981. Pollinator choice and stabilizing selection for flower color in *Delphinium nelsonii*. Evolution 35: 376–390. doi:10.1111/j.1558-5646.1981.tb04896.x 28563376

[mcag035-B85] Weeks EL, Frey FM. 2007. Seed production and insect visitation rates in *Hesperis matronalis* are not affected by floral symmetry. International Journal of Plant Sciences 168: 611–617. doi:10.1086/513483

