# Supplementary material for: Random and non-random variation in flower colour along an urban–rural gradient in the introduced mustard Hesperis matronalis
Source: Ann Bot. 2026 Feb 17;137(5):1290–304. doi: 10.1093/aob/mcag035 (PMC13197582; doi:10.1093/aob/mcag035)
Supplement: mcag035_Supplementary_Data [file mcag035_supplementary_data.zip › MaunderEtAl_Appendix_S3.docx]

**Appendix S3 – Analysis of colour morph diversity based on two morphs**

Here, we determine whether the results we obtained using a colour morph classification based on three morphs (white, pink, purple) hold when we use a 2-morph classification (light, dark). Because flowers we classified as pink were more variable in colour than those classified as white or purple (Appendix S2), we further classified, while in the field, pink flowers as light pink or dark pink. Hence, we can designate Dark flowers as either purple or dark pink, and light flowers as white or light pink. Shannon colour morph diversity based on two morphs correlated very strongly among stands with diversity based on 3 morphs for all three years (2021 *r* = + 0.87, 2022 *r* = +0.91, 2023 *r* = +0.93, all *P* < 0.0001).

As for the 3-morph analysis, there was wide variation in the frequencies of the two flower colour morphs among stands in all years. Two-morph colour diversity (*H*’_2_) varied across the full range of possible values (0–0.693) in 2021 (range = 0–0.693, mean = 0.51), 2022 (range = 0–0.693, mean = 0.51), and 2023 (range = 0–0.693, mean 0.52). *H*’_2_ also increased significantly and independently with both stand size (log_10_*N*, prediction 1) and human activity (NSB, prediction 2) in all years (Table S2) and the effects of these two predictors did not interact (all *P* > 0.28).

Among the 105 stands sampled in all three years, there was year to year consistency in 2-morph diversity, as *H*’_2_ corelated strongly between years (Kendall’s coefficient of concordance *W* = +0.76, *P* < 0.0001). The correlation between *H*’_2_ and NSB was consistently positive and did not vary among years. The correlation between *H*’_2_ and log_10_*N* varied significantly between years but was always positive (Table S3). *H*’_2_ did not vary among years.

The stands we sampled were usually polymorphic for flower colour (i.e. included both the dark and light morphs; 88.6% of 132 in 2021; 88.4% of 112 in 2022; 87.9% of 116 in 2023, Table S4). Monomorphic stands fixed for a single colour morph were about a tenth of the size of polymorphic stands and tended to be in areas with lower human activity (Table S5). As in the 3-morph analysis, monomorphic stands were almost always fixed for the darker morph (Table S4). Monomorphic dark-flowered stands made up 87% of 15 monomorphic stands in 2021, 92% of 13 in 2022, and 93% of 14 in 2023. This is significantly higher than would be expected based on the mean frequency of the dark morph in polymorphic stands (0.536 in 2021 χ^2^ = 6.59, simulated *P* = 0.014; 0.578 in 2022 χ^2^ = 6.35, *P* = 0.017; 0.561 in 2023 χ^2^ = 7.68, *P* = 0.0055). Moreover, all six stands that were monomorphic in all three years were fixed for the dark-flowered morph (Table S4).

In contrast to neutral expectations, logistic regression revealed that morph frequencies covaried with both human activity (NSB) and sometimes stand size (log_10_*N*) but these effects did not interact (Table S6). The dark morph decreased in frequency and the light morph increased in frequency with increasing human activity in all years. The frequencies of the dark morph decreased with stand size but only in 2022.

Including colour morph diversity predicted from this systematic variation in morph frequencies (*H’*_pred_) as a covariate in the linear models examining the effect of NSB and log_10_*N* on *H*’_2_ tended weakened the effect of both these predictors (Table S2). However, *H’*_pred_ was a significant predictor of *H*’_2_ only in 2021.

Table S2. Analyses of variation in colour morph diversity based on 2 morphs (*H*’_2_) among stands of *Hesperis matronalis*. In analysis A, variation in *H*’_2_ was fit to linear models with human activity (NSB), stand size (log_10_*N*) and their interaction as predictors (132 stands for 2021, 112 for 2022, 116 for 2023). Cells include *F*-tests of significance and standardized partial regression coefficients (*b*) when significant, or NA when that predictor was not included in that analysis. The effect of both human activity and stand size on *H*’_2_ (Fig. 2) might have been due to a systematic increase in the frequency of the dark morph in smaller stands and in areas with low human activity. In analysis B we tried to control for this effect by entering “predicted morph diversity” (*H’*_pred_) calculated from predicted morph frequencies (Fig. 3) as a covariate in the linear models. *H’*_pred_ was only a significant predictor of *H*’_2_ in 2021 but including it in the model as a covariate reduced the effect of both NSB and log_10_*N* for all years. However, predicted morph diversity correlated with both these other predictors, resulting in moderate variance inflation factors (range of VIF: 2021 = 1.22–1.55, 2022 = 1.42–2.05, 2023 = 1.18–2.03).

|  | A | | | B | | |
| --- | --- | --- | --- | --- | --- | --- |
| Year = | 2021 | 2022 | 2023 | 2021 | 2022 | 2023 |
| *r*^2^ = | 0.14 | 0.22 | 0.17 | 0.17 | 0.24 | 0.18 |
| Predictor |  |  |  |  |  |  |
| Human activity (NSB) | *b* = +0.206  *F*_1,129_ = 5.5  *P* = 0.020 | *b* = +0.231  *F*_1,109_ = 7.3  *P* = 0.0079 | *b* = +0.296  *F*_1,113_ = 11.7  *P* = 0.00088 | *b* = +0.155  *F*_1,129_ = 2.6  *P* = 0.084 | *b* = +0.322  *F*_1,109_ = 10.4  *P* = 0.0017 | *b* = +0.205  *F*_1,112_ = 3.2  *P* = 0.076 |
| Stand size (log_10_*N*) | *b* = +0.242  *F*_1,129_ = 7.7  *P* = 0.0064 | *b* = +0.379  *F*_1,109_ = 19.7  *P* < 0.0001 | *b* = +0.246  *F*_1,113_ = 8.1  *P* = 0.0053 | *b* = +0.129  *F*_1,129_ = 1.7  *P* = 0.19 | *b* = +0.481  *F*_1,109_ = 21.6  *P* < 0.0001 | *b* = +0.205  *F*_1,112_ = 4.8  *P* = 0.030 |
| NSB x log_10_*N* | –  *F*_1,128_ = 1.2 *P* = 0.28 | –  *F*_1,108_ = 0.16  *P* = 0.69 | –  *F*_1,112_ = 1.1  *P* = 0.30 | NA | NA | NA |
| Predicted morph diversity (*H’*_pred_) | NA | NA | NA | –  *F*_1,128_ = 5.4  *P* = 0.021 | *­*–  *F*_1,108_ = 2.9  *P* = 0.090 | *–*  *F*_1,112_ = 1.5  *P* = 0.23 |

Table S3. Analyses of variation in 2-morph colour morph diversity (*H*’_2_) among stands of *Hesperis matronalis.* This analysis involves the 105 stands that were sampled in all three years.

Variation in 2-morph colour diversity was fit to mixed-effects linear models with human activity (NSB), stand size (log_10_*N*) and year as fixed effects as well as 2-way interactions between year and both stand size and human activity. A 3-way interaction was not included because the interaction between human activity and stand size was not significant in either year (Table 1). Stand was included as a random effect. Models were fit using the *lmer* function in the lme4 R package (version 1.1-33, https://CRAN.R-project.org/package=lme4). Significance of fixed effects were evaluated using likelihood-ratio tests. Cells include tests of significance (χ^2^, *P*) and standardized partial regression coefficients (*b*) when significant. The effect of log_10_*N* varied slightly among years, with a stronger effect of log_10_*N* on *H*’_2_ in 2022 (*b* = +0.383) than in 2021 (*b* = +0.198) or 2023 (*b* = +0.174).

| Predictor |  |
| --- | --- |
| Human activity (NSB) | *b* = +0.331  χ^2^ = 12.5, *P* = 0.00041 |
| Stand size (log_10_*N*) | *b* = +0.054  χ^2^ = 10.2, *P* = 0.0013 |
| Year (Y) | χ ^2^ = 2.5, *P* = 0.29 |
| Y x NSB | χ^2^ = 1.0, *P* = 0.59 |
| Y x log_10_*N* | *b*_2022_ > *b*_2023_  χ^2^ = 13.9, *P* = 0.00094 |

Table S4. Variation in frequency of dark and light flower colour morphs in stands of *Hersperis matronalis* from eastern Ontario, Canada surveyed in three consecutive years (generations). Average morph frequency was calculated across all stands and from only stands polymorphic for flower colour. The bottom half of the table shows the numbers of stands fixed for each colour morph in each year and all years.

| Subset | Year | *n* stands | Morph frequency | |
| --- | --- | --- | --- | --- |
|  |  |  | Dark | Light |
| All stands | 2021 | 132 | 0.566 | 0.434 |
|  | 2022 | 112 | 0.618 | 0.382 |
|  | 2023 | 116 | 0.597 | 0.403 |
| Polymorphic stands | 2021 | 117 | 0.536 | 0.464 |
|  | 2022 | 99 | 0.578 | 0.422 |
|  | 2023 | 102 | 0.561 | 0.439 |
|  |  |  | Stands fixed for: | |
|  |  |  | Dark | Light |
| Monomorphic stands | 2021 | 15^1^ | 13 | 2 |
|  | 2022 | 13^2^ | 12 | 1 |
|  | 2023 | 14^3^ | 13 | 1 |
|  | All 3 years | 6^4^ | 6 | 0 |

Notes:

(1) Five of these stands were gone in 2022, two of which reappeared in 2023. Of the 11 resampled stands, four were polymorphic in future years, one just barely. Three stands had < 5 plants but five had > 30 plants.

(2) Seven of these stands were also fixed in 2021 (five of which stayed fixed in 2023). Three were polymorphic in 2021. One was located in 2021 but only became accessible in 2022 and remained fixed in 2023. Three stands had < 5 plants but five had > 30 plants.

(3) Ten of these stands were fixed in a previous year (five for all three years). Five were polymorphic in a previous year (including two that were gone in 2022). Two stands had < 5 plants but six had > 30 plants.

(4) The mean size of these stands across all three years ranged 19.3 – 144.0 and averaged 79.6.

Table S5. Comparison of stand size (log_10_*N*) and human activity (NSB) between stands of *Hesperis matronalis* monomorphic vs. polymorphic for flower colour. Monomorphic stands made up 14 of 132 stands in 2021, 10 of 112 stands in 2022 and 13 of 116 stands in 2023. Monomorphic and polymorphic stands were compared using Welch’s 2-sample *t*-test with a 1-tailed test of significance.

| Variable | Year | Monomorphic | Polymorphic | *t*-test (1-tailed *P*) |
| --- | --- | --- | --- | --- |
| Stand size  log_10_*N* | 2021 | 1.16 ± 0.16 | 2.12 ± 0.06 | *t* = 5.58, *P* < 0.0001 |
|  | 2022 | 1.29 ± 0.16 | 2.13 ± 0.06 | *t* = 4.93, *P* < 0.0001 |
|  | 2023 | 1.43 ± 0.20 | 2.27 ± 0.07 | *t* = 3.99, *P* = 0.00094 |
| Human activity  NSB | 2021 | –21.5 ± 0.13 | –20.9 ± 0.07 | *t* = 4.02, *P* = 0.00025 |
|  | 2022 | –21.4 ± 0.20 | –21.0 ± 0.08 | *t* = 2.2, *P* = 0.021 |
|  | 2023 | –21.6 ± 0.07 | –21.0 ± 0.08 | *t* = 5.6, *P* < 0.0001 |

Table S6. Analysis of variation in the frequencies of dark and light flowers among stands of *Hesperis matronalis* in eastern Ontario, Canada. The numbers of the two colour morphs sampled in each stand were modeled as a binomial response variable with human activity (NSB) and stand size (log_10_*N*) and their interaction as predictors using the *glmmTMB* function in the glmmTMB R package (version 1.1.11, https://github.com/glmmTMB/glmmTMB) with a betabinomial error distribution. The bottom half of the table reports the strength of regressions of observed frequencies of the dark morph on the dark frequencies predicted by the model.

| Year | 2021 | | 2022 | | 2023 | |
| --- | --- | --- | --- | --- | --- | --- |
| Multinomial regression | | | | | | |
| Predictor | LRT χ^2^ | *P* | LRT χ^2^ | *P* | LRT χ^2^ | *P* |
| Human activity (NSB) | 16.7 | < 0.0001 | 15.2 | < 0.0001 | 18.2 | < 0.0001 |
| Stand size (log_10_*N*) | 3.4 | 0.063 | 5.8 | 0.016 | 0.05 | 0.82 |
| NSB x log_10_*N* | 0.7 | 0.40 | 0.8 | 0.38 | 1.6 | 0.21 |
| Observed vs. predicted morph frequencies | | | | | | |
|  | *r*^2^ | *P* | *r*^2^ | *P* | *r*^2^ | *P* |
| Frequency of dark | 0.162 | < 0.0001 | 0.172 | < 0.0001 | 0.146 | < 0.0001 |
