# Supplementary material for: Random and non-random variation in flower colour along an urban–rural gradient in the introduced mustard Hesperis matronalis
Source: Ann Bot. 2026 Feb 17;137(5):1290–304. doi: 10.1093/aob/mcag035 (PMC13197582; doi:10.1093/aob/mcag035)
Supplement: mcag035_Supplementary_Data [file mcag035_supplementary_data.zip › MaunderEtAl_Appendix_S6.docx]

**Appendix S6 – Variation in colour morph diversity among generations**

Figure S5. Added-variable plots showing the relations between the standard deviation (SD) of flower colour morph diversity (*H’*) among generations and human activity (NSB) and the mean and standard deviation of stand size (log_10_*N*) across generations in *Hesperis matronalis* from eastern Ontario, Canada (105 stands sampled in all three years). Each point is a stand. The y-axis is residual SD of *H’* when the other two predictors are held constant. The x-axis is the residual of the focal predictor when the other two predictors are held constant.
