# Supplementary material for: Random and non-random variation in flower colour along an urban–rural gradient in the introduced mustard Hesperis matronalis
Source: Ann Bot. 2026 Feb 17;137(5):1290–304. doi: 10.1093/aob/mcag035 (PMC13197582; doi:10.1093/aob/mcag035)
Supplement: mcag035_Supplementary_Data [file mcag035_supplementary_data.zip › MaunderEtAl_Appendix_S7.docx]

**Appendix S7 – Distribution of stands with a high frequency of the purple morph and a spatial autocorrelation analysis**

Here we investigated whether there was clustering in the location of stands that were fixed for or had a high frequency of the purple morph. For simplicity the below figures and analysis were done using the data from 2021. Stands with a more even mix of the three colour morphs are located near the southern center of the study range - i.e. near the city center of Kingston, Ontario (Figure S6). Visually, stands with a very high frequency of the purple colour morphs are located along the top edges of the study range, which are also the locations with the lowest levels of human activity (Figure S6, see Figure S1 for NSB site values). The stands that are fixed for the purple morph have been highlighted in Figure S7 for further clarity. We ran a Global Moran’s I test for spatial autocorrelation with the moran.test() function and a permutation test with moran.mc() function from the spdep package in R. This was performed looking at frequency of the purple morph as the variable of interest and we used distance-based weighing with a bandwidth of 5 km. There was significant positive spatial clustering (I = 0.33, P < 0.01). Since human activity levels are correlated with distance from the city center of Kingston, it is not surprising that there is some global spatial autocorrelation, given the urban-rural cline in flower colour that we report in this paper. We also performed a local Moran’s I test. The output for sites with P values less than 0.05 can are listed in Table S9 and the significant clusters and outliers can be seen in Figure S7. Four of the 12 stands fixed for the purple morph appear to be part of a positively spatially correlated cluster (Figure S8). It is possible that these four stands may be the results of a previous introduction from a source with a high frequency of the purple morph. However, a majority of the stands fixed for the purple morph (8 out of 12 stands) are not significantly spatially autocorrelated (Figure S8).

Figure S6. Location of sites surveyed in 2021 (n = 132), with pie charts displaying the frequency of each colour morph (W = white, P = pink, V = purple). Stands with a high frequency of the purple morphs are often located around the edges of the study region.

Figure S7: Map of sites surveyed in 2021 (n = 132). Purple coloured circles highlight the location of the twelve stands monomorphic for purple. The grey circles represent all other sites.

Figure S8. Map highlighting sites that are significant spatial clusters or outliers based on the results of a Local Moran’s I test (n = 132). Red circles indicate sites that are High-High clusters, meaning that sites with high purple morph frequency are found near other sites with a high frequency. Blue circles indicate sites that are Low-High outliers, meaning these sites have a low frequency of the purple morph despite being in an area with a higher frequency of the purple morph. There are four red circles at the top of this figure (one quite hidden) that represent four stands that are all fixed for the purple morph and seems to be clustered together.

Table S9. Output of local Moran’s I test investigating spatial autocorrelation between sites in the frequency of purple morph. Distance-based weighing was used with a bandwidth of 5 km. Only the output for significant sites (P < 0.05) is listed. A positive I value indicates spatial clustering (e.g. sites with high purple morph frequency are found near other sites with high frequency) and negative I values indicate outliers (e.g. sites with a low frequency of the purple morph are located near sites with a higher frequency of the purple morph). E.Ii is the expected Local Moran’s I under no spatial autocorrelation, Var.Ii is the variance, and Z.Ii is the standardize Local Moran’s I.

| **sitecode** | **Ii** | **E.Ii** | **Var.Ii** | **Z.Ii** | **p.value** |
| --- | --- | --- | --- | --- | --- |
| AD-1 | 0.185 | -0.001 | 0.007 | 2.253 | 0.024 |
| AD-3 | 0.992 | -0.009 | 0.202 | 2.228 | 0.026 |
| AD-4 | 0.839 | -0.009 | 0.185 | 1.972 | 0.049 |
| AD-5 | 0.289 | -0.001 | 0.017 | 2.244 | 0.025 |
| BR-10 | -0.776 | -0.006 | 0.143 | -2.037 | 0.042 |
| BR-17 | -2.173 | -0.030 | 0.927 | -2.226 | 0.026 |
| CL-6 | 1.933 | -0.022 | 0.926 | 2.031 | 0.042 |
| CL-7 | 2.033 | -0.022 | 1.032 | 2.023 | 0.043 |
| DEV-2 | 1.650 | -0.022 | 0.407 | 2.621 | 0.009 |
| DEV-4 | 1.812 | -0.022 | 0.464 | 2.694 | 0.007 |
| FR-1 | 0.289 | 0.000 | 0.011 | 2.824 | 0.005 |
| FR-2 | 1.485 | -0.017 | 0.284 | 2.817 | 0.005 |
| FR-4 | 1.112 | -0.009 | 0.177 | 2.665 | 0.008 |
| FR-8 | 1.268 | -0.011 | 0.231 | 2.661 | 0.008 |
| HWY10-10 | 1.246 | -0.007 | 0.332 | 2.174 | 0.030 |
| HWY33-13 | 0.645 | -0.003 | 0.031 | 3.659 | 0.000 |
| HWY33-14 | 0.601 | -0.003 | 0.081 | 2.120 | 0.034 |
| HWY33-2 | -0.370 | -0.002 | 0.016 | -2.870 | 0.004 |
| HWY33-4 | 0.363 | -0.002 | 0.009 | 3.746 | 0.000 |
| HWY33-5 | 1.004 | -0.019 | 0.106 | 3.142 | 0.002 |
| HWY33-6 | 0.304 | -0.001 | 0.006 | 3.876 | 0.000 |
| HWY33-7 | 1.357 | -0.024 | 0.269 | 2.665 | 0.008 |
| HWY38-11 | -0.434 | -0.001 | 0.031 | -2.465 | 0.014 |
| LP-1 | 1.264 | -0.011 | 0.100 | 4.029 | 0.000 |
| LP-2 | 1.632 | -0.022 | 0.197 | 3.731 | 0.000 |
| LP-3 | 1.108 | -0.011 | 0.072 | 4.179 | 0.000 |
| MR-1 | 0.895 | -0.015 | 0.193 | 2.070 | 0.038 |
